# Molecular subtyping of European swine influenza viruses and scaling to high-throughput analysis

**DOI:** 10.1186/s12985-018-0920-z

**Published:** 2018-01-10

**Authors:** Emilie Bonin, Stéphane Quéguiner, Cédric Woudstra, Stéphane Gorin, Nicolas Barbier, Timm C. Harder, Patrick Fach, Séverine Hervé, Gaëlle Simon

**Affiliations:** 1 ANSES, Ploufragan-Plouzané Laboratory, Swine Virology Immunology Unit, National Reference Laboratory for Swine Influenza, Ploufragan, France; 2 Bretagne Loire University, Rennes, France; 3 ANSES, Laboratory for Food Safety, IdentyPath Platform, Maisons-Alfort, France; 4 Institute of Diagnostic Virology, Friedrich-Loeffler Institute, Greifswald-Insel Riems, Germany; 5 Current address: INRA, US 1426, GeT-PlaGe, Genotoul, Castanet-Tolosan, France

**Keywords:** Influenzavirus, Subtyping, Hemagglutinin, Neuraminidase, Pig, High-throughput real-time RT-PCR, LightCycler®1536, Surveillance, Diagnosis

## Abstract

**Background:**

Swine influenza is a respiratory infection of pigs that may have a significant economic impact in affected herds and pose a threat to the human population since swine influenza A viruses (swIAVs) are zoonotic pathogens. Due to the increasing genetic diversity of swIAVs and because novel reassortants or variants may become enzootic or have zoonotic implications, surveillance is strongly encouraged. Therefore, diagnostic tests and advanced technologies able to identify the circulating strains rapidly are critically important.

**Results:**

Several reverse transcription real-time PCR assays (RT-qPCRs) were developed to subtype European swIAVs in clinical samples previously identified as containing IAV genome. The RT-qPCRs aimed to discriminate HA genes of four H1 genetic lineages (H1_av_, H1_hu_, H1_huΔ146–147_, H1pdm) and one H3 lineage, and NA genes of two N1 lineages (N1, N1pdm) and one N2 lineage. After individual validation, each RT-qPCR was adapted to high-throughput analyses in parallel to the amplification of the IAV M gene (target for IAV detection) and the β-actin gene (as an internal control), in order to test the ten target genes simultaneously on a large number of clinical samples, using low volumes of reagents and RNA extracts.

**Conclusion:**

The RT-qPCRs dedicated to IAV molecular subtyping enabled the identification of swIAVs from the four viral subtypes that are known to be enzootic in European pigs, i.e. H1_av_N1, H1_hu_N2, H3N2 and H1N1pdm. They also made it possible to discriminate a new antigenic variant (H1_hu_N2_Δ146–147_) among H1_hu_N2 viruses, as well as reassortant viruses, such as H1_hu_N1 or H1_av_N2 for example, and virus mixtures. These PCR techniques exhibited a gain in sensitivity as compared to end-point RT-PCRs, enabling the characterization of biological samples with low genetic loads, with considerable time saving. Adaptation to high-throughput analyses appeared effective, both in terms of specificity and sensitivity. This new development opens novel perspectives in diagnostic capacities that could be very useful for swIAV surveillance and large-scale epidemiological studies.

**Electronic supplementary material:**

The online version of this article (10.1186/s12985-018-0920-z) contains supplementary material, which is available to authorized users.

## Background

Swine influenza (SI) is a highly contagious viral respiratory infection of pigs that has become enzootic in areas densely populated with this species [1]. It is responsible for significant economic losses in affected herds due to morbidity, stunted growth and secondary infections. Swine influenza A viruses (swIAVs) are also of public health concern due to their zoonotic potential. Although human infections remain subclinical or commonly produce only mild symptoms, swIAVs may be responsible for fatal cases and/or contribute, e.g. by reassortment, to pandemics, as illustrated by the pandemic that occurred in 2009 [2, 3].

swIAVs are enveloped viruses belonging to the genus Influenzavirus A, family *Orthomyxoviridae*. Their genome consists of eight segments of single-stranded RNA of negative polarity. They are typed according to their major antigenic determinants, i.e. the surface glycoproteins hemagglutinin (HA) and neuraminidase (NA) [4]. SwIAVs can evolve by antigenic drift (nucleotide point mutations, substitutions, deletions or insertions in HA and NA) and/or by antigenic shift (HA and/or NA reassortments) [5, 6]. Three subtypes of swIAVs, H1N1, H3N2 and H1N2, are simultaneously circulating among pigs worldwide, whereas lineages may vary within each subtype depending on the region, i.e. North America, Europe and Asia [7].

In Europe, the predominant H1N1 viruses are entirely of avian origin, introduced from wild ducks to pigs in 1979 and referred as “avian-like” swine H1N1 (H1_av_N1) [8, 9], with HA genes belonging to the clade 1C defined recently [10]. H3N2 viruses became widespread in European pigs in the mid-1980s. They are “human-like reassortant swine H3N2” viruses that combine the HA and NA genes from a descendant of the 1968 Hong Kong pandemic H3N2 virus and the internal genes of the H1_av_N1 virus [11]. The dominant H1N2 viruses are “human-like reassortant swine H1N2” (H1_hu_N2) viruses that emerged in 1994. They retained the genotype of European H3N2 swIAVs, except for the HA gene that they acquired from a human H1N1 virus from the 1980s [12] and which belongs to clade 1B [10]. Thus, these three swIAV lineages share common internal genes, but have clearly distinguishable HA- and NA- encoded genes [8]. Since 2009, the H1N1 pandemic (H1N1pdm) virus has been transmitted from humans to pigs and has become a fourth enzootic swIAV lineage in European pigs [8]. The H1N1pdm virus originates from a reassortment between a North American triple reassortant swIAV and a Eurasian H1_av_N1 swIAV [13]. The H1pdm gene (clade 1A [10]) and the N1pdm gene, can be distinguished genetically from H1 and N1 genes encountered in other European swIAVs [9]. Due to co-circulation and co-infections, reassortant viruses originating from these four European enzootic lineages are also sporadically but regularly detected in several European countries, i.e. H1_av_N2, H1_hu_N1, H1pdmN2, and some of them have even become enzootic in certain regions [8, 14, 15]. Moreover, a new variant resulting from an antigenic drift in the HA gene of the enzootic H1_hu_N2 virus has been identified on several occasions over the last few years in Italy [16, 17] and France, where it was named H1_hu_N2_Δ146–147_ [18].

Due to the increasing genetic diversity of swIAVs in Europe and because novel reassortants or variants may become enzootic in swine or have zoonotic implications, surveillance is strongly encouraged [8]. Therefore, diagnostic tests and advanced technologies able to identify the circulating strains rapidly are critically important. The development and harmonization of diagnostic tools for detection (M gene) and subtyping (HA and NA genes) of European swIAVs have been initiated in some European countries, especially through the concerted action “European surveillance network for influenza in pigs (ESNIP) 3” [8, 19]. One issue, following a multi-center study using a panel of reference strains from several countries, was the selection of the best-performing primers/probe sets and amplification protocols that could be used in reverse transcription real-time PCR assays (RT-qPCRs) for the detection of HA and NA genes from the different genetic lineages [20]. In this paper, we describe RT-qPCRs aimed specifically at identifying HA and NA genes from European swIAVs, taking into account local strains isolated recently in France. Following an original approach, we also report their adaptation to high-throughput analyses, in parallel to the amplification of the IAV M gene and the β-actin gene, which provide a comprehensive tool for detection and subtyping of European swIAVs in large biological sample sets.

## Methods

### Clinical samples and virus strains

Nasal swabs (MW950Sent2mL Virocult®, Kitvia, Labarthe-Inard, France) were collected from herds in France by veterinary practitioners or ANSES personnel, in the context of virological diagnosis passive surveillance programs or specific epidemiological investigations. They were taken from pigs during outbreaks of acute respiratory disease. The swabs were mixed vigorously and supernatants were stored at −70 °C until analysis. Some lung samples were also obtained from necropsy of fatal cases. Total viral RNAs were extracted from 200 μL of nasal swab supernatants or 20–30 mg of lung homogenate using the NucleoSpin RNA or Nucleospin 8 RNA kits (Macherey-Nagel, Hoerdt, France) or the RNeasy Mini Kit (Qiagen, Courtaboeuf, France). IAV genome detection was performed by M gene RT-qPCR using one of the two ready-to-use commercial kits previously validated by the French National Reference Laboratory (NRL), i.e. the LSI VetMAX™ Swine Influenza A-A/H1N1/2009- included kit (Life technologies, Carlsbad, CA, USA) or the ADIAVET™ SIV REALTIME kit (Bio-X Diagnostics, Rochefort, Belgique). Both steps, i.e. viral RNA purification and IAV genome detection, followed the instructions provided by RT-qPCR manufacturers [21].

Virus isolation was attempted from positive clinical samples in Madin Darby canine kidney (MDCK) cells, according to standard procedures [22]. Reference strains, previously selected as representative of the main viral subtypes and lineages encountered in European pigs over the last few years, were also retrieved from the collection of the French NRL or kindly provided by the European surveillance network for influenza in pigs (ESNIP) [8, 23]. A/California/04/2009 (H1N1pdm) was obtained from the French National Reference Center for Influenza, Institut Pasteur, Paris.

### Initial subtyping

#### Molecular subtyping

Initially, swIAV-positive samples and/or virus isolates were subjected to molecular subtyping using conventional RT-PCR assays, as previously described [19, 23]. One multiplex RT-PCR enabled the specific detection of HA genes of the H1_av_ (clade 1C [10]), H1_hu_ (clade 1B.1 [10]) and H3 lineages, while another enabled the amplification of NA genes of the N1 and N2 lineages. Since 2010, H1 and N1 genes from H1N1pdm have been screened using ready-to-use commercial kits previously validated by the French NRL for Swine Influenza [21], according to the manufacturer’s instructions. The H1pdm gene (clade 1A.3.3.2 [10]) was detected using the real-time RT-PCR LSI VetMAX™ Swine Influenza A-A/H1N1/2009-H1 detection kit (Life technologies, Carlsbad, CA, USA) or the ADIAVET™ A/H1N1(2009) REALTIME kit (Bio-X Diagnostics, Rochefort, Belgique). The N1pdm gene was detected using the real-time RT-PCR LSI VetMAX™ Swine Influenza A-A/H1N1/2009-N1 detection kit (Life technologies, Carlsbad, CA, USA).

#### Antigenic subtyping

Some virus strains were further propagated in 9-day-old specific pathogen-free (SPF) embryonated chicken eggs and subjected to antigenic characterization using hemagglutination inhibition (HI) tests, according to standard procedures [22]. Allantoic fluids were tested with hyperimmune sera produced in SPF pigs against reference strains representative of the different subtypes: A/Sw/Cotes d’Armor/0388/2009 (H1_av_N1), A/Sw/Flandres/1/1998 (H3N2), A/sw/Scotland/410440/94 (H1_hu_N2), A/Sw/Cotes d’Armor/113/2006 (H1_hu_N2), A/Sw/Cotes d’Armor/0070/2010 (H1_hu_N1), A/Sw/Cotes d’Armor/0186/2010 (H1_av_N2), A/Sw/Sarthe/0255/2010 (H1N1pdm) and A/Sw/France/22–130212/2013 (H1_hu_N2_Δ146–147_) [23, 24].

#### Sequencing

The subtype and lineage of some selected swIAVs and some positive field samples were determined and/or confirmed by sequencing the HA and NA gene segments. The viral genome was reverse transcribed using universal primers [25] and SuperScript II Reverse Transcriptase (Life technologies, Carlsbad, CA, USA) following the manufacturer’s instructions. Full length HA and NA genes were amplified using Platinum Taq DNA Polymerase High Fidelity (Life technologies, Carlsbad, CA, USA) and in-house designed primers (sequences available on request). Amplified products were then separated in agarose gel and purified with the QIAquick Gel Extraction Kit (Qiagen, Hilden, Germany). Both strands of the amplicons were sequenced with the same primers used for the amplification, as well as with additional internal primers (sequences available on request). Sequencing reactions were performed using the BigDye Terminator v3.1 Cycle Sequencing Kit (Life technologies, Carlsbad, CA, USA) on an automatic DNA sequencer ABI 3130 Genetic Analyzer (Life technologies, Carlsbad, CA, USA). Manual editing of sequences and assembly refinement were done with Vector NTI Advance 11.0 software (Life technologies, Carlsbad, CA, USA). The Influenza Research Database (IRD) was screened with BLASTN2 to identify sequences closely related to the HA and NA sequences.

### Primers and probes for HA and NA RT-qPCRs

Sets of primers (forward and reverse) and the probe were designed to specifically amplify the different HA and NA genes from European swIAVs, on the basis of alignments of nucleotide sequences of viruses isolated since 2000 and retrieved from the IRD, GISAID (Global Initiative on Sharing All Influenza Data) or Genbank databases. Melting temperatures and/or basic properties of oligonucleotides were approximated using OligoCalc [26] or Vector NTI Advance^®^ sequence analysis and design software (Thermo Fischer Scientific, Waltham, MA, USA) (Table 1). Whereas ready-to-use commercial kits have previously been validated for H1pdm and N1pdm detection [21], new sets of primers and the probe specific to these two genes were designed in our study in order to include H1pdm and N1pdm RT-qPCRs in the high-throughput PCR array. Thus, the H1_av_, H1_hu_, H1pdm and H3 sets specifically amplify HA genes from the European H1_av_N1, H1_hu_N2, H1N1pdm and H3N2 lineages, respectively. The N1 set matches with NA genes from both the H1_av_N1 and the H1N1pdm lineages whereas the N1pdm set specifically amplifies the NA gene from the H1N1pdm lineage. The N2 primers and probe can detect NA genes from both the H1_hu_N2 and the H3N2 lineages. In addition, an H1_huΔ146–147_ set was manually designed to identify the novel antigenic variant that have emerged among H1_hu_N2 viruses following antigenic drift [18]. Alignments comprised full length HA sequences of H1_hu_N2 and H1_hu_N2_Δ146–147_, as well as various H1_hu_N1 reassortant viruses. Thus, the H1_huΔ146–147_ set design took into account a deletion of 6 nucleotides and a mutation located in the receptor binding site of the H1_hu_N2_Δ146–147_ variant (manuscript in preparation). Finally, M and β-actin sets were included, since in-house RT-qPCRs for swIAV and reference gene detection were used, respectively, as controls in high-throughput PCR assays. All RT-qPCRs used standard TaqMan DNA probes except those for H1pdm, N1pdm and H1_huΔ146–147_ which used highly specific DNA probes with conjugated minor groove binder (MGB) groups at the 3‘-end. The fluorophore covalently attached to the 5’-end of the oligonucleotide probe was 6-carboxyfluorescein (FAM), hexachlorofluorescein (HEX) or cyanine5 (Cy5) (Table 1). All standard TaqMan probes used black hole quencher (BHQ1) at the 3′-end. Primers and labelled standard TaqMan probes were purchased from Sigma-Aldrich (Saint-Louis, MO, USA). MGB-labelled probes were from Life technologies (Carlsbad, CA, USA).

**Table 1:** Primers and probes

Target Gene	Primer/Probe	Sequence, labeling	Location^a^	Reference Sequence (accession number)
H1_av_	H1av_Fo	gaaggrggatggacaggaatga	1063–1084	A/Sw/Cotes d’Armor/0388/2009 (KC881265)
	H1av_Re	caattahtgarttcactttgttgc	1178–1201	
	H1av_Pr	(HEX)-tctggttacgcagcwgatcagaaaa-(BHQ1)	1126–1150	
H1_hu_	H1hu_Fo_1	gagggggrtggaccggaatgatagatgga[i]5tggttatcatca	1090–1109	A/Sw/Cotes d’Armor/0113/2006 (AM503902)
	H1hu_Fo_2	ggatggtacggttatcatca	1064–1109	
	H1hu_Re_1	acctacagctgtgaattgagtgttcatyttntcg[i]5agagttcacct	1184–1204	
	H1hu_Re_2	tttcgatcacagaattcacct	1184–1233	
	H1hu_Pr	(FAM)-cagggatctggctatgctgcagayc-(BHQ1)	1120–1144	
H1_huΔ146–147_	H1hu_dif_Fw	agttcagtatcatcattcgagagattcgaaat	367–398	A/Sw/France/22-130212/2013 (KJ128323)
	H1hu_dif_Rv	actgcatcatgctcccataagggga	468–492	
	H1hu_var_FAM	(FAM)-cagcataggagca-(MGB)	454–466	
H1_pdm_	H1pdm_Fo	gggcattcaccatccatctact	582–603	A/California/04/2009 (FJ966082)
	H1pdm_Re	cctcactttgggtcttattgctattt	689–714	
	H1pdm_Pr	(FAM)-atacagcaagaagttcaagc-(MGB)	666–685	
H3	H3_Swine_Fw	cttgatggrgmaaaytgcaca	223–243	A/Sw//France/59–120031/2012 (KC345622)
	H3_Swine_Rv	ggcacatcatawgggtaaca	337–356	
	H3_Swine_CY5 (or HEX)	(CY5 or HEX)-ctctattgggrgaccctcaytgtga-(BHQ1)	254–278	
N1	N1.3_F	agrccttgyttctgggttga	1255–1274	A/Sw/Germany/SIV04/2008 (FN429079) [20]
	N1.3_R	accgtctggccaagacca	1363–1380	
	AIV N1.3 FAM	(FAM)-atytggacyagtgggagcagcat-(BHQ1)	1306–13,028	
N1_pdm_	N1pdm_Fo	gggacagacaataacttctcaataaagc	1144–1171	A/California/04/2009 (FJ966082)
	N1pdm_Re	ttcagcatccagaactaacagggt	1220–1243	
	N1pdm_Pr	(FAM)-aaatgagtggtcaggatat-(MGB)	1188–1206	
N2	N2_1367F	agtctggtggacytcaaayag	1305–1325	A/Sw/Bakum/8602/1999 (EF409258) [20]
	N2_1468R	ttgcgaaagcttatatagvcatga	1397–1420	
	AIV N2 1444 HEX	(HEX)-ccatcaggccatgagcctgwwccata-(BHQ1)	1357–1382	
M	SIV-Forw	agatgagtcytctaaccgaggtcg	24–47	A/Sw/France/22–130212/2013 (KM267912) [30]
	SIV-Rev	tgcaaaracayyttcmagtctctg	101–124	
	M64_FAM	(FAM)-tcaggccccctcaaagccga-(BHQ1)	74–93	
β-actin	Sw_actine_Forw	ctcgatcatgaagtgcgacgt		[31]
	Sw_actine_Rev	gtgatctccttctgcatcctgtc		
	β-actine_sus_scrofa_HEX	(HEX)-atcaggaaggacctctacgccaacacgg-(BHQ1)		

^a^From the first nucleotide of the coding sequence, except for M (from the first nucleotide of the segment)

### Real-time RT-PCR assays for European swIAV subtyping

Similarly to virus detection by M gene RT-qPCR, the HA/NA subtyping methods were assessed on RNA extracted from amplified viruses or clinical samples using the NucleoSpin RNA kit (Macherey-Nagel, Hoerdt, France) or the RNeasy Mini Kit (Qiagen, Courtaboeuf, France) following the manufacturer’s instructions. Real-time RT-PCR assays were run either on Chromo4 (Bio-Rad Laboratories, Hercules, CA, USA) or MX3005P (Stratagene, Agilent Technologies, La Jolla, CA, USA). Each RT-qPCR mixture contained 5 μL of RNA extract and 20 μL of master mix containing 1X GoTaq Probe qPCR Master Mix, dUTP (2X) (Promega, Fitchburg, WI, USA), 2X GoScript RT Mix for 1-step RT-qPCR (50X) (Promega, Fitchburg, WI, USA) as well as primers and probe in different concentrations depending on the target gene. Thus, the master mix contained 800 nM of each primer and 100 nM of probe for H1_av_, H3, N1 and N2, 800 nM of each primer and 140 nM of probe for H1_hu_, 400 nM of each primer and 500 nM of probe for H1_huΔ146–147_, and 800 nM of each primer and 250 nM of probe for H1pdm and N1pdm. All RT-qPCRs were run as singleplex assays, except N1 and N2 that were run as a duplex assay. The cycling conditions used for H1_av_, H1_hu_, H3 and N1/N2 were: 15 min reverse transcription at 45 °C, 2 min of denaturation at 95 °C and 42 cycles of 15 s at 95 °C and 1 min at 56 °C. The program used for H1_huΔ146–147_, H1pdm and N1pdm (with MGB probe) was 15 min reverse transcription at 45 °C, 2 min of denaturation at 95 °C, and 40 cycles of 15 s at 95 °C and 1 min at 60 °C.

### Evaluation of real-time RT-PCR subtyping performance

Several panels incorporating virus strains and/or clinical samples were constituted to evaluate the performance of the RT-qPCRs developed for the amplification of the H1_av_, H1_hu_, H1_huΔ146–147_, H3, N1 and N2 genes.

A first panel (panel 1) of 42 swIAV isolates was set up to assess the analytical specificity of the RT-qPCRs designed for H1_av_, H1_hu_, H3, N1 and N2 gene amplifications. Among these virus strains, 32 belong to one of the four European enzootic lineages, i.e. 12 H1_av_N1, 10 H1_hu_N2, 8 H1N1pdm and 2 H3N2, whereas 10 others are isolates originating from reassortments between enzootic viruses, i.e. 5 H1_hu_N1 and 5 H1_av_N2 (Table 2). RNA extracts were diluted in RNAse free-water to adjust the quantification cycle (Cq) value to 24–27 when tested with one of the M gene RT-qPCRs described above, in order to reduce the risk of cross-reactions between H1_av_ and H1_hu_.

**Table 2:** Specificity of the real-time RT-PCR assays developed for the identification of swIAV HA and NA genes originating from the European enzootic lineages H1_av_N1, H1_hu_N2, H1N1pdm and H3N2

Virus strains (Panel 1)				M gene RT-qPCR^b^ (Cq- value)	Real-time RT-PCRs for molecular subtyping^c^ (Cq-value)				
Subtype and lineage			Name		HA subtyping			NA subtyping	
HA lineage of the most antigenically related reference strain (from HI tests)	Lineage known from conventional RT-PCRs and commercial H1pdm and N1pdm RT-qPCRs^b^								
	HA	NA^a^			H1_av_	H1_hu_	H3	N1	N2
H1_av_	H1_av_	N1_av_	A/Sw/Aisne/0054/10	25.48	24.16	no Cq	no Cq	24.61	no Cq
			A/Sw/Ille et Vilaine/0187/11	26.87	26.92	no Cq	no Cq	24.45	no Cq
			A/Sw/Ille et Vilaine/0208/11	24.6	23.45	no Cq	no Cq	19.05	no Cq
			A/Sw/Morbihan/0294/11	27	25.88	no Cq	no Cq	24.86	no Cq
			A/Sw/Finistere/0307/11	27.12	30.23	no Cq	no Cq	28.64	no Cq
			A/Sw/LoireAtlantique/0405/11	26.9	29.84	no Cq	no Cq	28.03	no Cq
			A/Sw/France/41–120137/12	24.33	19.84	no Cq	no Cq	27.55	no Cq
			A/Sw/France/72–120183/12	25.12	25.16	no Cq	no Cq	25.27	no Cq
			A/Sw/France/29–120326/12	26.34	36.15	no Cq	no Cq	27.68	no Cq
			A/Sw/France/22–120340/12	24.29	23.40	no Cq	no Cq	22.70	no Cq
			A/Sw/France/56–120452/12	25.75	30.63	no Cq	no Cq	25.91	no Cq
			A/Sw/France/53–130065/13	26.65	33.03	no Cq	no Cq	30.16	no Cq
H1_av_	H1_av_	N2	A/Sw/Cotes d’Armor/0102/08	25.38	23.13	no Cq	no Cq	no Cq	23.62
			A/Sw/Cotes d’Armor/0186/10	26.56	24.92	no Cq	no Cq	no Cq	26.69
			A/Sw/Morbihan/0213/11	24.26	24.37	no Cq	no Cq	no Cq	23.98
			A/Sw/Morbihan/0599/11	24.6	26.17	no Cq	no Cq	no Cq	24.06
			A/Sw/France/37–120,345/12	24.34	27.26	no Cq	no Cq	no Cq	24.91
H1_hu_	H1_hu_	N2	A/Sw/Cotes d’Armor/0074/11	26.81	no Cq	30.80	no Cq	no Cq	29.33
			A/Sw/France/22–110,153/11	26.87	no Cq	32.06	no Cq	no Cq	28.08
			A/Sw/Finistere/0186/11	26.63	no Cq	31.08	no Cq	no Cq	29.74
			A/Sw/Ille et Vilaine/0346/11	25.6	no Cq	26.83	no Cq	no Cq	25.26
			A/Sw/Ille et Vilaine/0415/11	24.05	no Cq	28.07	no Cq	no Cq	26.43
			A/Sw/Maine et Loire/0589/11	24.65	no Cq	23.19	no Cq	no Cq	23.27
			A/Sw/France/56–120177/12	25.05	no Cq	30.97	no Cq	no Cq	30.66
			A/Sw/France/22–120255/12	25.68	no Cq	29.93	no Cq	no Cq	27.52
			A/Sw/France/29–120,258/12	27.17	no Cq	31.01	no Cq	no Cq	29.21
			A/Sw/France/22–130032/13	24.54	no Cq	23.30	no Cq	no Cq	25.82
H1_hu_	H1_hu_	N1_av_	A/Sw/Cotes d’Armor/0046/08	25.85	no Cq	28.06	no Cq	24.46	no Cq
			A/Sw/Cotes d’Armor/0070/10	24.59	no Cq	28.80	no Cq	24.54	no Cq
			A/Sw/Morbihan/0163/10	26.12	no Cq	32.22	no Cq	26.53	no Cq
			A/Sw/France/56–110525/10	25.63	no Cq	29.24	no Cq	32.04	no Cq
			A/Sw/France/22–120067/12	26.46	no Cq	31.69	no Cq	26.66	no Cq
H1_pdm_	H1_pdm_	N1_pdm_	A/California/04/09	25.92	no Cq	no Cq	no Cq	27.76	no Cq
			A/Sw/Sarthe/0255/10	26.26	no Cq	no Cq	no Cq	26.51	no Cq
			A/Sw/Sarthe/0262/10	25.32	no Cq	no Cq	no Cq	26.02	no Cq
			A/Sw/Cotes d’Armor/110466/10	25.15	no Cq	no Cq	no Cq	27.35	no Cq
			A/Sw/Haute-Loire/0578/11	26.42	no Cq	no Cq	no Cq	28.44	no Cq
			A/Sw/France/18–120158/12	27.29	no Cq	no Cq	no Cq	29.73	no Cq
			A/Sw/France/18–120333/12	24.04	no Cq	no Cq	no Cq	24.37	no Cq
			A/Sw/France/71–130116/13	24.68	no Cq	no Cq	no Cq	28.93	no Cq
H3	H3	N2	A/Sw/Flandres/1/98	24.98	no Cq	no Cq	24.39	no Cq	25.58
			A/Sw/France/59–120031/12	26.1	no Cq	no Cq	27.47	no Cq	26.68

^a^N1_av_ means that the conventional N1 RT-qPCR was positive but the N1pdm commercial RT-qPCR was negative; ^b^Commercial kits [21]; ^c^HA RT-qPCRs were run as monoplex procedures whereas NA RT-qPCRs were run in duplex; Cq: quantification cycle

Panel 2 was assembled to test the specificity of the H1_huΔ146–147_ gene RT-qPCR, i.e. its ability to differentiate the H1 gene of the antigenic variant H1_hu_N2_Δ146–147_ virus among HA genes previously identified as belonging to the H1_hu_ lineage. This panel consisted of RNA extracts obtained from 46 swIAV strains previously identified as H1_hu_N2 viruses (44/46) or H1_hu_N1 viruses (2/46) (Additional file 1).

The analytical sensitivity of the assays was evaluated by testing 10-fold serial dilutions (from 10^−2^ to 10^−9^) of RNA extracted from reference strains (Table 3). The range of linearity, the coefficient of linear regression (R^2^) and the efficacy were then calculated to evaluate the performance (Table 4). The repeatability of each RT-qPCR was estimated by testing, in 5–6 independent assays, the RNA extract dilution immediately below the last dilution in which the target gene was detected by the assay, and calculating the inter-assay coefficient of variation (CV) (Table 5).

**Table 3:** Analytical sensitivity of the real-time RT-PCRs for H1_av_, H1_hu,_ H3, N1 and N2 on ten-fold dilutions of RNA extracted from swIAVs of different subtypes

Virus strain		Virus stock dilution	M gene RT-qPCR^a^(Cq-value)	Real-time RT-PCR^b^ for molecular subtyping (Cq-value)					
Lineage	Name								
				H1_av_	H1_hu_	H1_huΔ146–147_	H3	N1	N2
H1_av_N1	A/Sw/Cotes d’Armor/0388/2009	10^−1^	12.52	16.10	no Cq	nt	no Cq	15.52	no Cq
		10^−2^	16.09	19.52	no Cq	nt	no Cq	18.75	no Cq
		10^−3^	20.16	22.77	no Cq	nt	no Cq	22.46	no Cq
		10^−4^	23.83	25.85	no Cq	nt	no Cq	25.71	no Cq
		10^−5^	**27.03**	28.86	no Cq	nt	no Cq	**28.18**	no Cq
		10^−6^	29.79	32.32	no Cq	nt	no Cq	no Cq	no Cq
		10^−7^	**33.36**	**36.26**	no Cq	nt	no Cq	no Cq	no Cq
		10^−8^	38.59	no Cq	no Cq	nt	no Cq	no Cq	no Cq
		10^−9^	no Cq	no Cq	no Cq	nt	no Cq	no Cq	no Cq
		10^−10^	no Cq	no Cq	no Cq	nt	no Cq	no Cq	no Cq
H1_hu_N2	A/Sw/Scotland/410440/1994	10^−1^	13.47	no Cq	15.18	nt	no Cq	no Cq	16.90
		10^−2^	17.15	no Cq	18.53	nt	no Cq	no Cq	19.92
		10^−3^	20.43	no Cq	21.99	nt	no Cq	no Cq	23.54
		10^−4^	**23.79**	no Cq	**25.19**	nt	no Cq	no Cq	**26.60**
		10^−5^	27.25	no Cq	no Cq	nt	no Cq	no Cq	no Cq
		10^−6^	30.11	no Cq	no Cq	nt	no Cq	no Cq	no Cq
		10^−7^	33.05	no Cq	no Cq	nt	no Cq	no Cq	no Cq
		10^−8^	37.48	no Cq	no Cq	nt	no Cq	no Cq	no Cq
		10^−9^	no Cq	no Cq	no Cq	nt	no Cq	no Cq	no Cq
		10^−10^	no Cq	no Cq	no Cq	nt	no Cq	no Cq	no Cq
H1_hu_N2_Δ146–147_	A/Sw/France/22–130212/2013	10^−1^	10.98	nt	11.88	12.68	nt	no Cq	11.48
		10^−2^	14.67	nt	15.26	16.10	nt	no Cq	14.38
		10^−3^	17.82	nt	18.66	20.07	nt	no Cq	17.78
		10^−4^	21.3	nt	21.77	22.95	nt	no Cq	20.92
		10^−5^	25.64	nt	25.03	26.53	nt	no Cq	24.54
		10^−6^	**28.06**	nt	no Cq	29.83	nt	no Cq	33.03*
		10^−7^	31.54	nt	no Cq	33.36	nt	no Cq	no Cq
		10^−8^	**35.16**	nt	no Cq	37.03	nt	no Cq	no Cq
		10^−9^	no Cq	nt	no Cq	**39.63**	nt	no Cq	no Cq
		10^−10^	no Cq	nt	no Cq	no Cq	nt	no Cq	no Cq
H3N2	A/Sw/Flandres/1/1998	10^−1^	13.13	no Cq	no Cq	nt	17.045	no Cq	16.865
		10^−2^	16.7	no Cq	no Cq	nt	19.88	no Cq	19.725
		10^−3^	20.21	no Cq	no Cq	nt	23.55	no Cq	23.275
		10^−4^	**23.57**	no Cq	no Cq	nt	27.075	no Cq	26.67*
		10^−5^	26.9	no Cq	no Cq	nt	30.235	no Cq	no Cq
		10^−6^	30.07	no Cq	no Cq	nt	33.53	no Cq	no Cq
		10^−7^	32.7	no Cq	no Cq	nt	36.335	no Cq	no Cq
		10^−8^	**37.04**	no Cq	no Cq	nt	37.86	no Cq	no Cq
		10^−9^	no Cq	no Cq	no Cq	nt	**39.99**	no Cq	no Cq
		10^−10^	no Cq	no Cq	no Cq	nt	no Cq	no Cq	no Cq

*Cq* quantification cycle, *nt* not tested. M gene RT-qPCRs were run on one replicate whereas molecular subtyping RT-qPCRs were run on duplicates (Thermocycler MxPro – Mx3005P). Thus, Cq-values for molecular subtyping RT-qPCRs are mean Cq-values between duplicates, except numbers indicated in italics with an asterisk (*). Cq-values indicated in bold represent detection limits. ^a^Commercial kits [21]; ^b^HA RT-qPCRs were run as simplex assays; N1 and N2 RT-qPCRs were run in duplex

**Table 4:** Performance of real-time RT-PCRs for H1_av_, H1_hu,_ H1_huΔ146–147_, H3, N1 and N2 on ten-fold dilutions of RNA extracted from swIAVs representative of the different subtypes

Virus strain	Criteria	Real-time RT-PCR^b^ for molecular subtyping					
		H1_av_	H1_hu_	H1_hu Δ146–147_	H3	N1	N2
A/Sw/Cotes d’Armor/0388/2009 (H1_av_N1)	Range of linearity^a^	< 12.52 to 33.36				< 12.52 to 27.03	
	Slope	−3.2918				−3.226	
	R^2^	0.9986				0.9957	
	Efficacy	101.27%				104.16%	
A/Sw/Scotland/410440/1994 (H1_hu_N2)	Range of linearity^a^		<13.47 to 23.79				<13.47 to 23.79
	Slope		−3.3483				−3.2725
	R^2^		0.9998				0.9957
	Efficacy		98.91%				102.10%
A/Sw/France/22–130212/2013 (H1_hu_N2_Δ146–147_)	Range of linearity^a^		<10.98 to 25.64	<10.98 to >35.16			<10.98 to 28.06
	Slope		−3.281	−3.4008			−3.266
	R^2^		0.9997	0.9992			0.9988
	Efficacy		101.74%	96.81%			102.39%
A/Sw/Flandres/1/1998 (H3N2)	Range of linearity^a^				16.7 to 32.7		<13.13 to 23.57
	Slope				−3.2964		−3.297
	R^2^				0.9982		0.998
	Efficacy				101.08%		101.07%

^a^The range of linearity is given as the interval of Cq-values obtained from M gene RT-qPCR on diluted samples (Thermocycler MxPro – Mx3005P). R^2^: coefficient of linear regression. ^b^HA RT-qPCRs were run as simplex assays; N1 and N2 RT-qPCRs were run in duplex

**Table 5:** Repeatability and intermediate precision of real-time RT-PCRs for H1_av_, H1_hu,_ H1_huΔ146–147,_ H3, N1 and N2 amplifications

Real-time RT-PCR^a^ for molecular subtyping	RNA extract from (virus strain – name and subtype)	RNA extract dilution (≈ M gene Cq-value)	Mean Cq-value	SD	Inter-assay CV (%)
H1_av_	A/Sw/Cotes d’Armor/0388/2009 (H1_av_N1)	10^−4^ (≈ 23.83)	24.50	0.9	1.20
H1_hu_	A/Sw/Scotland/410440/1994 (H1_hu_N2)	10^−3^ (≈ 20.43)	25.63	0.39	1.52
H1_hu Δ146–147_	A/Sw/France/22–130212/2013 (H1_hu_N2_Δ146–147_)	10^−4^ (≈ 21.3)	22.80	0.71	3.12
H3	A/Sw/Flandres/1/1998 (H3N2)	10^−2^ (≈ 16.7)	18.60	0.31	1.68
N1	A/Sw/Cotes d’Armor/0388/2009 (H1_av_N1)	10^−4^ (≈ 23.83)	25.64	0.37	1.46
N2	A/Sw/Scotland/410440/1994 (H1_hu_N2)	10^−3^ (≈ 20.43)	24.78	1.19	4.79
	A/Sw/Flandres/1/1998 (H3N2)	10^−3^ (≈ 20.21)*	28.56	1.86	6.51

^a^HA RT-qPCRs were run as simplex assays; N1 and N2 RT-qPCRs were run in duplex. *Cq* quantification threshold, *SD* standard deviation, *CV* coefficient of variation. Each RNA extract was tested (either on Thermocycler MxPro – Mx3005P or Chromo4) in 6 different assays at the dilution corresponding to 10 times the detection limit of the concerned RT-qPCR (except H3 that was run on a lowest dilution of the H3N2 strain). The RNA extract dilution marked with an asterisk (*) was tested in 5 independent assays

Panels 3 and 4 were set up to evaluate the diagnostic abilities of the various RT-qPCRs, i.e. their potential to identify HA and NA genes directly from clinical samples previously shown to contain swIAV genome (M gene RT-qPCR positive). Panel 3 included 60 RNA extracts obtained from 59 nasal swab supernatants and one lung tissue homogenate (Table 6). The swIAV genome was previously subtyped in 37 nasal swabs and the lung sample (11 samples were positive for H1_av_N1, 4 for H1_av_N2, 15 for H1_hu_N2, 2 for H1_hu_N1, 1 for H3N2, 4 for H1N1pdm, and 1 for both H1_av_ and H1_hu_ genes mixed with N2), whereas in 22 nasal swab supernatants the IAV genome was only partially subtyped using conventional RT-PCRs (18 samples tested positive for H1_av_ but not for N1 or N2; 3 samples tested negative for any HA but were found positive for N2, and 1 sample was found to be negative for HA and NA). Panel 4 was specifically assembled to assess the diagnostic ability of the H1_huΔ146–147_ RT-qPCR. It was composed of 78 RNA extracts, i.e. 56 obtained from nasal swab supernatants and 22 from swIAV strains, that were previously found either positive for H1_hu_ but not sequenced, or positive for N2 but in which HA was not identified, or in which neither HA nor NA were identified (see Additional file 2).

**Table 6:** Performance of real-time RT-PCR assays targeting the H1_av_, H1_hu_, H3, N1 and N2 genes in porcine clinical samples containing European swIAVs

Clinical samples^a^ (Panel 3)			M gene RT-qPCR (Cq-value)	Real-time RT-PCRs for molecular subtyping^c^ (Cq-value)				
swIAV lineage known from conventional RT-PCRs and commercial H1N1pdm RT-qPCRs		Identification		HA subtyping			NA subtyping	
HA	NA^b^			H1_av_	H1_hu_	H3	N1	N2
H1_av_	N1_av_	120137–4	20.6	19.19	no Cq	no Cq	19.85	no Cq
		120183–5	18.1	19.33	no Cq	no Cq	19.25	no Cq
		130004–5	17.59	19.08	no Cq	no Cq	17.75	no Cq
		130020–4	26.43	31.38	no Cq	no Cq	26.83	no Cq
		130028–3	26.43	22.13	no Cq	no Cq	26.98	no Cq
		130065–5	17	23.08	no Cq	no Cq	20.73	no Cq
		130144–4	22.26	22.20	no Cq	no Cq	22.46	no Cq
		130153–6	22.46	32.33	no Cq	no Cq	22.49	no Cq
		130157–6	27.46	24.22	no Cq	no Cq	24.25	no Cq
		140014–4	17.56	nt	nt	nt	17.45	no Cq
		140016–5	19.09	nt	nt	nt	19.73	no Cq
H1_av_	N2	120057–3	17.89	17.25	no Cq	no Cq	no Cq	19.72
		120345–2	17.51	21.51	no Cq	no Cq	no Cq	20.35
		130133–3	21.18	21.67	no Cq	no Cq	no Cq	23.54
		110214–1	28.92	27.82	no Cq	no Cq	nt	nt
H1_hu_	N2	100231-1^c^	18.57	no Cq	24.79	no Cq	no Cq	21.86
		120037–4	23.88	no Cq	27.79	no Cq	no Cq	27.04
		120285–6	23.37	no Cq	25.58	no Cq	no Cq	24.45
		120424–5	21.32	no Cq	25.43	no Cq	no Cq	24.96
		130039–2	22.5	no Cq	22.66	no Cq	no Cq	22.08
		130111–5	21	no Cq	22.79	no Cq	no Cq	22.06
		130111–6	19.6	nt	nt	nt	no Cq	22.04
		130129–4	18.29	no Cq	26.63	no Cq	no Cq	22.58
		130193–6	21.95	no Cq	25.36	no Cq	no Cq	24.11
		130129–5	17.86	no Cq	27.63	nt	no Cq	22.63
		130140–5	13.74	no Cq	20.21	nt	no Cq	16.36
		130285–4	29.54	nt	nt	nt	no Cq	27.57
		130420–4	22.31	no Cq	23.90	nt	no Cq	24.12
		130429–6	22.42	no Cq	24.26	nt	no Cq	21.40
		130431–4	15.82	no Cq	18.31	nt	no Cq	17.41
H1_hu_	N1_av_	110619–8	17.28	no Cq	21.86	no Cq	18.74	no Cq
		120067–3	27.47	no Cq	33.08	no Cq	27.47	no Cq
H1_av_/H1_hu_	N2	130103–2	21.46	28.36	23.42	no Cq	**22.72**	24.56
H1_pdm_	N1_pdm_	110578–1	22.16	no Cq	no Cq	no Cq	24.09	no Cq
		120158–7	16.97	no Cq	no Cq	no Cq	19.37	no Cq
		120333–1	20.08	no Cq	no Cq	no Cq	21.70	no Cq
		130116–1	25.21	no Cq	no Cq	no Cq	28.01	no Cq
H3	N2	120031–1	21.83	no Cq	no Cq	22.68	no Cq	24.46
H1_av_	N?	130044–2	32.18	no Cq	no Cq	nt	**30.37**	no Cq
		130113–2	33.24	no Cq	no Cq	nt	no Cq	no Cq
		130120–3	33.91	41.43	no Cq	nt	**31.64**	no Cq
		130135–6	30.77	28.13	no Cq	nt	**27.85**	no Cq
		130175–2	34.36	no Cq	no Cq	nt	no Cq	no Cq
		130185–6	27.41	no Cq	no Cq	nt	**24.76**	no Cq
		130188–3	30.5	27.73	no Cq	nt	**28.30**	no Cq
		130236–1	32.88	no Cq	no Cq	nt	no Cq	no Cq
		130259–2	33.26	32.05	no Cq	nt	**31.22**	no Cq
		130282–4	27.53	37.19	no Cq	nt	**27.45**	no Cq
		130392–3	31.53	34.52	no Cq	nt	**32.07**	no Cq
		130300–4	31.22	no Cq	no Cq	nt	no Cq	no Cq
		130319–5	34.2	no Cq	no Cq	nt	no Cq	no Cq
		130336–4	33.7	no Cq	no Cq	nt	no Cq	no Cq
		130387–5	23.93	28.20	no Cq	nt	**25.70**	no Cq
		130391–4	34.75	no Cq	no Cq	nt	no Cq	no Cq
		130419–6	27.43	no Cq	no Cq	nt	**28.53**	no Cq
		130439–5	29.97	no Cq	no Cq	nt	no Cq	no Cq
H?	N2	130023–4	25.43	no Cq	**27.58**	nt	no Cq	26.27
		130034–3	23.05	no Cq	**26.51**	nt	no Cq	25.68
		130277–3	22	nt	nt	nt	no Cq	24.52
nt	nt	130133–5	23.02	**22.90**	**31.53**	nt	no Cq	**24.76**

^a^All samples were nasal swab supernatants except the marked one that was a lung sample. ^b^N1_av_ means that the conventional N1 RT-PCR was positive but the N1pdm commercial RT-qPCR was negative. ^C^all RT-qPCRs were run as monoplex procedures except N1 and N2 that were run in a duplex. Cq: quantification cycle; nt = not tested. Bold data correspond to additional results obtained with the novel RT-qPCRs as compared to conventional RT-PCRs

### High-throughput real-time RT-PCR procedure

High-throughput real-time RT-PCR amplifications (M, β-actin, H1_av_, H1_hu_, H1_huΔ146–147,_ H1pdm, H3, N1, N1pdm and N2) were conducted on a LightCycler^®^1536 real-time PCR system (Roche, Meylan, France). A Bravo automated liquid handling platform equipped with a chiller and a PlateLoc thermal microplate sealer (Agilent Technologies, La Jolla, CA, USA) was used as a microplate dispenser. Each RT-qPCR mixture contained 1 μL of RNA extract and 1 μL of master mix containing 1X RealTime ready DNA Probes Master (Roche, Meylan, France), 2X GoScript RT Mix for 1-step RT-qPCR (50X) (Promega, Fitchburg, WI, USA), 800 nM of each concerned primer, with the exception of the M gene forward primer that was included at a final concentration of 400 nM, and 250 nM of standard or MGB-labelled TaqMan probe. The one-step real-time RT-PCR program involved a 30 min reverse transcription of RNA at 45 °C, followed by a 2 min denaturation step at 95 °C, and lastly 40 cycles of 0 s (i.e. a pulse) at 95 °C and 1 min at 58 °C.

### Evaluation of high-throughput RT-qPCR performance

Panel 5 was established to evaluate the analytical specificity of the real-time RT-PCRs (including new in-house H1pdm and N1pdm RT-qPCRs) when using the high-throughput RT-qPCR procedure (see Additional file 3). This panel included of RNA extracts obtained from 88 swIAVs and 30 nasal swab supernatants. The isolates as well as the viruses present in clinical samples were first subtyped using RT-qPCRs run under low-throughput conditions. Serial 10-fold dilutions (10^−1^ to 10^−8^) of RNA obtained from 8 reference strains were prepared in water to evaluate the analytical sensitivity of each RT-qPCR when run on the LightCycler^®^1536 (Table 7). The M gene copy number present in each dilution point was measured using a duplex M/β-actin gene RT-qPCR as described separately [27]. Each dilution point was tested in duplicate.

**Table 7:** Analytical sensitivity of the real-time RT-PCRs for M, H1_av_, H1_hu,_ H1_huΔ146–147_, H1pdm, H3, N1, N1pdm and N2 on ten-fold dilutions of RNA extracted from swIAVs of different subtypes when run on LightCycler^®^1536

Virus strain		Virus stock dilution	M gene RT-qPCR (Cq-value)	Real-time RT-PCR for molecular subtyping (Cq-value)							
Lineage	Name										
				H1_av_	H1_hu_	H1_huΔ146–147_	H1pdm	H3	N1	N1pdm	N2
H1_av_N1	A/Sw/Cotes d’Armor/0388/2009	10^−1^	12.01	12.53	no Cq	no Cq	no Cq	no Cq	12.33	no Cq	no Cq
		10^−2^	14.90	15.80	no Cq	no Cq	no Cq	no Cq	16.02	no Cq	no Cq
		10^−3^	19.46	19.84	no Cq	no Cq	no Cq	no Cq	20.63	no Cq	no Cq
		10^−4^	22.83	23.14	no Cq	no Cq	no Cq	no Cq	23.77	no Cq	no Cq
		10^−5^	26.53	26.85	no Cq	no Cq	no Cq	no Cq	27.11	no Cq	no Cq
		10^−6^	29.52	29.72	no Cq	no Cq	no Cq	no Cq	30.24	no Cq	no Cq
		10^−7^	32.81	32.51*	no Cq	no Cq	no Cq	no Cq	33.74*	no Cq	no Cq
		10^−8^	35.45	no Cq	no Cq	no Cq	no Cq	no Cq	35.34*	no Cq	no Cq
H1_av_N2	A/Sw/Cotes d’Armor/0186/2010	10^−1^	15.01	15.14	no Cq	no Cq	no Cq	no Cq	no Cq	no Cq	14.32
		10^−2^	18.14	18.43	no Cq	no Cq	no Cq	no Cq	no Cq	no Cq	18.04
		10^−3^	21.72	21.96	no Cq	no Cq	no Cq	no Cq	no Cq	no Cq	22.34
		10^−4^	25.34	25.27	no Cq	no Cq	no Cq	no Cq	no Cq	no Cq	26.12
		10^−5^	28.91	27.95	no Cq	no Cq	no Cq	no Cq	no Cq	no Cq	29.51
		10^−6^	32.76	31.62	no Cq	no Cq	no Cq	no Cq	no Cq	no Cq	32.40
		10^−7^	no Cq	no Cq	no Cq	no Cq	no Cq	no Cq	no Cq	no Cq	34.31*
		10^−8^	no Cq	no Cq	no Cq	no Cq	no Cq	no Cq	no Cq	no Cq	no Cq
H1_hu_N2	A/Sw/Cotes d’Armor/0113/2006	10^−1^	14.52	no Cq	16.11	no Cq	no Cq	no Cq	no Cq	no Cq	13.71
		10^−2^	17.45	no Cq	19.33	no Cq	no Cq	no Cq	no Cq	no Cq	17.68
		10^−3^	22.48	no Cq	23.92	no Cq	no Cq	no Cq	no Cq	no Cq	22.71
		10^−4^	25.87	no Cq	27.60	no Cq	no Cq	no Cq	no Cq	no Cq	26.40
		10^−5^	28.49	no Cq	30.10*	no Cq	no Cq	no Cq	no Cq	no Cq	28.89
		10^−6^	33.49	no Cq	33.58*	no Cq	no Cq	no Cq	no Cq	no Cq	32.04*
		10^−7^	no Cq	no Cq	no Cq	no Cq	no Cq	no Cq	no Cq	no Cq	34.76
		10^−8^	no Cq	no Cq	no Cq	no Cq	no Cq	no Cq	no Cq	no Cq	no Cq
H1_hu_N2_Δ146–147_	A/Sw/France/22–130212/2013	10^−1^	8.59	no Cq	11.90	10.61	no Cq	no Cq	no Cq	no Cq	8.58
		10^−2^	12.08	no Cq	15.01	13.69	no Cq	no Cq	no Cq	no Cq	13.32
		10^−3^	15.54	no Cq	18.64	18.07	no Cq	no Cq	no Cq	no Cq	16.69
		10^−4^	19.05	no Cq	22.40	21.91	no Cq	no Cq	no Cq	no Cq	20.91
		10^−5^	22.85	no Cq	25.88	26.00	no Cq	no Cq	no Cq	no Cq	24.98*
		10^−6^	25.44	no Cq	29.72	29.21	no Cq	no Cq	no Cq	no Cq	27.95
		10^−7^	29.78	no Cq	32.37*	32.17*	no Cq	no Cq	no Cq	no Cq	31.09
		10^−8^	32.73	no Cq	no Cq	36.31	no Cq	no Cq	no Cq	no Cq	34.43
H1_hu_N1	A/Sw/Cotes d’Armor/0070/2010	10^−1^	14.85	no Cq	18.39	no Cq	no Cq	no Cq	16.85	no Cq	no Cq
		10^−2^	18.11	no Cq	21.66	no Cq	no Cq	no Cq	20.45	no Cq	no Cq
		10^−3^	22.90	no Cq	26.08	no Cq	no Cq	no Cq	25.07	no Cq	no Cq
		10^−4^	25.71	no Cq	28.73	no Cq	no Cq	no Cq	27.57	no Cq	no Cq
		10^−5^	29.68	no Cq	31.39	no Cq	no Cq	no Cq	30.95	no Cq	no Cq
		10^−6^	no Cq	no Cq	no Cq	no Cq	no Cq	no Cq	34.42	no Cq	no Cq
		10^−7^	33.47*	no Cq	no Cq	no Cq	no Cq	no Cq	no Cq	no Cq	no Cq
		10^−8^	No Cq	no Cq	no Cq	no Cq	no Cq	no Cq	no Cq	no Cq	no Cq
H1_hu_N1_Δ147_	A/Sw/Cotes d’Armor/0190/2006	10^−1^	13.49	no Cq	16.90	no Cq	no Cq	no Cq	16.07	no Cq	no Cq
		10^−2^	17.24	no Cq	20.87	no Cq	no Cq	no Cq	19.93	no Cq	no Cq
		10^−3^	20.86	no Cq	23.92	no Cq	no Cq	no Cq	23.79	no Cq	no Cq
		10^−4^	25.01	no Cq	28.30	no Cq	no Cq	no Cq	27.43	no Cq	no Cq
		10^−5^	28.21	no Cq	no Cq	no Cq	no Cq	no Cq	30.37	no Cq	no Cq
		10^−6^	31.31	no Cq	no Cq	no Cq	no Cq	no Cq	34.50*	no Cq	no Cq
		10^−7^	no Cq	no Cq	no Cq	no Cq	no Cq	no Cq	35.28*	no Cq	no Cq
		10^−8^	no Cq	no Cq	no Cq	no Cq	no Cq	no Cq	no Cq	no Cq	no Cq
H1N1pdm	A/Sw/Sarthe/0255/2010	10^−1^	13.31	no Cq	no Cq	no Cq	19.30	no Cq	17.24	16.38	no Cq
		10^−2^	16.62	no Cq	no Cq	no Cq	22.49	no Cq	21.07	20.55	no Cq
		10^−3^	20.44	no Cq	no Cq	no Cq	26.63	no Cq	24.76	23.80	no Cq
		10^−4^	24.65	no Cq	no Cq	no Cq	30.63	no Cq	28.82	27.93	no Cq
		10^−5^	27.94	no Cq	no Cq	no Cq	33.24	no Cq	32.08	30.39	no Cq
		10^−6^	31.88	no Cq	no Cq	no Cq	36.13*	no Cq	33.85*	34.77	no Cq
		10^−7^	33.50*	no Cq	no Cq	no Cq	no Cq	no Cq	no Cq	no Cq	no Cq
		10^−8^	no Cq	no Cq	no Cq	no Cq	no Cq	no Cq	no Cq	no Cq	no Cq
H3N2	A/Sw/Flandres/1/1998	10^−1^	12.96	no Cq	no Cq	no Cq	no Cq	13.28	no Cq	no Cq	13.23
		10^−2^	16.61	no Cq	no Cq	no Cq	no Cq	17.15	no Cq	no Cq	16.51
		10^−3^	20.88	no Cq	no Cq	no Cq	no Cq	21.32	no Cq	no Cq	21.98
		10^−4^	24.05	no Cq	no Cq	no Cq	no Cq	24.44	no Cq	no Cq	25.42
		10^−5^	27.57	no Cq	no Cq	no Cq	no Cq	28.15	no Cq	no Cq	28.67
		10^−6^	30.48	no Cq	no Cq	no Cq	no Cq	32.06	no Cq	no Cq	32.54
		10^−7^	34.01*	no Cq	no Cq	no Cq	no Cq	33.74	no Cq	no Cq	no Cq
		10^−8^	35.31*	no Cq	no Cq	no Cq	no Cq	34.38*	no Cq	no Cq	no Cq

*Cq* quantification cycle. Cq-values are mean Cq-values between duplicates except numbers indicated with an asterisk that were obtained from a single sample (*). Cq-values highlighted in grey represent detection limits

## Results

### Analytical performances of the real-time RT-PCRs for H1_av_, H1_hu_, H1_huΔ146–147_, H3, N1 and N2 gene amplification

The analytical performances of the novel RT-qPCRs developed for European swIAV subtyping in addition to the H1pdm and N1pdm commercial kits previously validated were first evaluated in low-throughput analyses.

#### Analytical specificity

Analyses of 42 swIAV isolates from panel 1 showed perfect concordance between the results from H1_av_, H1_hu_, H3, N1 and N2 RT-qPCRs and those expected from conventional, i.e. end-point RT-PCRs and/or sequencing data, indicating 100% analytical specificity for the assays (Table 2). Despite non negligible viral loads, i.e. Cq-values around 25 in M gene RT-qPCR, none of these assays showed any undesired amplification of genes from influenza viruses other than those expected. According to plan, the N1 RT-qPCR was able to detect NA genes from the H1_av_N1 and H1N1pdm lineages, while the N2 RT-qPCR amplified NA genes of viruses from the H1_hu_N2 and H3N2 lineages.

Among the 46 swIAV strains included in panel 2 and previously identified as viruses that bear an H1_hu_ gene, 25/44 H1_hu_N2 strains were detected by the H1_huΔ146–147_ RT-qPCR indicating they were variant viruses, in accordance with HA gene sequencing (see Additional file 1). All other H1_hu_N2 strains did not exhibit the 6 nt-deletion encountered in the variant and were not detected by specifically designed RT-qPCR H1_huΔ146–147,_ showing that there were no false-positive results. By contrast, the two H1_hu_N1 viruses were not detected even though they also showed the 146–147 amino acid deletion, due to a mutation in the MGB probe target region. Indeed, sequencing revealed that these viruses retained a thymine residue (T) in probe position 12, instead of having mutated into a cytosine residue (C) as compared to the parental H1_hu_ gene. These reassortant viruses, 100% identical in their HA genes, were isolated from the same farm 2.5 months apart.

#### Analytical sensitivity

Real-time RT-PCRs developed for HA or NA subtyping of previously detected swIAVs exhibited lower sensitivity levels than the M gene assays used for the detection, except those amplifying the H1_huΔ146–147_ and the H3 genes (Table 3). Thus, assays conducted on 10-fold dilutions of reference strains indicated that the detection limits based on M gene RT-qPCR Cq-values would be >35 for H1_huΔ146–147_ and H3, around 33 for H1_av_, around 27 for N1, between 24 and 28 for N2 depending on the virus subtype, and around 24 for H1_hu_. Based on calculation of the ranges of linearity, it appeared that all genes were correctly detected from the first (10^−1^) strain dilution, i.e. at Cq-value <13.5, except the H3 gene for which the lowest limit was estimated at the second (10^−2^) dilution, at a Cq-value equivalent to 16.7 (Table 4). Between the limits of the range of linearity, the slopes of the standard curves varied from −3.3483 to −3.266. The coefficients of linear regression (R^2^) varied from 0.9957 to 0.9998 and the efficiencies were calculated between 96.81% and 104.16%. Repeatability and intermediate precision, assessed by calculating the inter-assay CVs, gave satisfactory results (Table 5). The inter-assay CVs were all <10%, ranging from 1.20 for the H1_av_ RT-qPCR to 6.51 for the N2 RT-qPCR run on the H3N2 strain.

### Diagnostic abilities of the RT-qPCR assays targeting the H1_av_, H1_hu_, H1_huΔ146–147_, H3, N1 and N2 genes of European swIAVs

Nasal swabs from animals naturally infected with H1_av_N1, H1_hu_N2 or H3N2 swIAVs, as well as with H1_av_N2 or H1_hu_N1 reassortant viruses (panel 3), were detected positive for both HA and NA corresponding genes in 100% of cases tested for both genes (Table 6). Additional samples tested either only in the simplex HA assays or in the N1/N2 multiplex assay were also qualified positive according to the expected HA or NA gene. Inclusion of 4 samples positive for H1N1pdm confirmed N1 amplification without any H1_av_ gene amplification. Sample 130103–2, which was previously known to contain both the H1_av_ and H1_hu_ genes together with the N2 gene, was also proved here to contain the N1 gene, enabling us to confirm the hypothesis of a mixture of H1_av_N1 and H1_hu_N2 viruses in this sample. Analyses of 18 samples previously partially subtyped as “H1_av_N?” confirmed the H1_av_ subtype in 9/18 cases. Others did not give a positive signal, probably due to limiting genome amounts (7/11 were samples with M gene RT-qPCR Cq-values >32, while the H1_av_ RT-qPCR detection limit was estimated around M gene Cq-value = 33). Conversely, the N1 gene was identified in 10/18 H1_av_N? samples, even in samples where M gene Cq-value >27, which was estimated as the detection limit. The N2 gene was not amplified in any of these samples. Panel 3 also comprised 3 H?N2 samples. The N2 gene was detected in all of them, whereas the H1_hu_ gene was identified in 2/3. The last one (sample 130277–3) was shown to have an H1_hu_ gene after propagation in cell culture (data not shown). Finally, a sample (130133–5) that was left fully unsubtyped using conventional RT-PCRs was here classified as containing the H1_av_, H1_hu_ and N2 genes, confirming the ability to detect virus mixtures using these RT-qPCRs.

The diagnostic ability of the H1_huΔ146–147_ RT-qPCR was evaluated by testing 78 samples of mainly H1N2 or H?N2 subtypes included in panel 4. The H1_huΔ146–147_ gene was identified in 3/15 H1_hu_N2 isolates (not sequenced previously), in 16/46 nasal swab supernatants in which the H1_hu_N2 virus was identified, as well as in 1/6 H1_hu_N? samples (Additional file 2). Although the specificity of the H1_huΔ146–147_ was previously demonstrated, these results were further validated by sequencing the HA genes of the three isolates that tested positive for the H1_huΔ146–147_ gene as well as the HA gene of strain A/Sw/France/29–150034/15 that tested negative. Sequencing confirmed that the three positive strains contained the 6 nt deletion by contrast to the other one that contained a H1_hu_ gene without this deletion (Genbank accession numbers KJ128334, KY241154, KY241155 and KY241115). Thus, 20/78 viruses or clinical samples previously identified as H1_hu_-positive were rapidly identified as having an H1_huΔ146–147_ gene.

### Analytical performances of the HA and NA RT-qPCRs in high-throughput analyses

Real-time RT-PCRs for H1_av_, H1_hu_, H1_huΔ146–147_, H3, N1 and N2 gene amplifications, as well as RT-qPCRs designed to amplify the H1pdm and N1pdm genes specifically, were evaluated in high-throughput analyses. Real-time RT-PCRs targeting the IAV M gene and the porcine β-actin genes were included in order to check the amount and quality of the genetic material and/or demonstrate any potential PCR inhibition. These last RT-qPCRs were previously validated in low-throughput analyses [21, 27]. Thus, 10 RT-qPCR assays were run together on a LightCycler®1536 to analyze the 118 samples from panel 5, i.e. 83 swIAVs, 5 virus mixtures and 30 nasal swab supernatants, all previously analyzed in low-throughput PCR assays (see Additional file 3). As expected, all samples were detected positive for the M gene. RNA extracts from virus strains exhibited M gene Cq-values ranging from <5 to 16.69, whereas extracts from clinical samples exhibited higher Cq-values, which mostly ranged between 14 and 30. Only 2/118 samples (1.7%) (both RNA extracts from MDCK cell culture supernatants) were found negative for the β-actin gene.

All HA and NA RT-qPCRs exhibited 100% specificity, as no undesirable cross-reaction or false-positive results were obtained, irrespective of the nature of the sample, i.e. RNA extracts from virus stock containing high amounts of genetic material, or RNA extracts from nasal swab supernatants. As expected, the H1_av_ RT-qPCR amplified H1 genes originating from the H1_av_N1 lineage, i.e. H1 genes from H1_av_N1 enzootic viruses and from H1_av_N2 reassortants. The H1_hu_ RT-PCR amplified H1 genes from H1_hu_N2 viruses, H1_hu_N2_Δ146–147_ variants and H1_hu_N1 reassortants. By contrast, the H1_huΔ146–147_ RT-PCR only detected the H1_hu_N2_Δ146–147_ antigenic variants. In accordance with the results obtained in low-throughput analyses, it did not detect H1_hu_N1 reassortants bearing H1_hu_ genes with one or two amino acid deletions close to the receptor-binding site (RBS) (called H1_hu_N1_Δ146–147_ and H1_hu_N1_Δ147_). The H1pdm RT-qPCR amplified HA genes of viruses from the H1N1pdm lineage but also any other H1 gene, and the H3 RT-qPCR amplified HA from H3N2 viruses only. The N1 RT-qPCR amplified N1 genes from both the H1_av_N1 and the H1N1pdm lineages, whereas the N1pdm RT-qPCR amplified the N1 genes from H1N1pdm viruses specifically. The N2 RT-qPCR amplified NA genes from all HxN2 viruses. Thus, all samples were fully subtyped according to expected results, including the virus mixtures, except one of them in which the N2 gene was not amplified in parallel to the H1_av_, H1_hu_ and N1 genes, as expected. As observed for the M gene, the Cq-values obtained for the HA and NA RT-qPCRs were lower on RNAs extracted from virus isolates as compared to clinical samples. In several cases, the Cq-value was too low to be precisely determined (< 5). A sample without any viral RNA but rather consisting of water was added in each PCR array as a negative control and no unspecific detection was observed (data not shown).

In order to go further in evaluating the sensitivity of the methods when run as high-throughput analyses, each RT-qPCR (except the one targeting the β-actin gene) was tested against 10-fold serial dilutions (10^−1^ to 10^−8^) of reference strains (Table 7). Depending on the virus stock and its initial genomic load, the M gene was still detected to the last dilution or to the 10^−6^ or the 10^−7^ dilution. In all cases, it was detected to the 10^−5^ dilution. The H1_av_, H1_huΔ146–147_ and H1pdm genes were detected in samples exhibiting a corresponding M gene Cq-value until 32, approximately, while the H3 gene detection limit was found to exceed an M gene Cq-value >35. The detection limit for the H1_hu_ gene varied from 25 to 33 depending on the virus (parental virus, antigenic variant, reassortant or reassortant with deletion), that of N1 from 29 to 31, and that of N2 from 30 to 33. The N1pdm was detected until an M gene Cq-value of 31. The ranges of linearity are given in Table 8. The slopes calculated over these ranges were all (except one) comprised between −3.2 and −3.7, which means the efficacies of the RT-PCRs varied from 85% to 105%. The N2 RT-qPCR run on the reference H3N2 strain was the only one found to be outside these limits, showing an efficacy of 80.51%.

**Table 8:** Performance of real-time RT-PCRs for M, H1_av_, H1_hu,_ H1_huΔ146–147_, H1pdm, H3, N1, N1pdm and N2 on ten-fold dilutions of RNA extracted from swIAVs of different subtypes when run on thermocycler LightCycler^®^1536

Virus strain	Criteria	M gene RT-qPCR^a^	Real-time RT-PCR for molecular subtyping							
			H1_av_	H1_hu_	H1_hu Δ146–147_	H1pdm	H3	N1	N1pdm	N2
A/Sw/Cotes d’Armor/0388/2009 (H1_av_N1)	Range of linearity^a^	<12.01 to >35.45	<12.01 to 32.81					<12.01 to >35.45		
	Slope	−3.423	−3.386					−3.356		
	R^2^	0.9963	0.9971					0.9914		
	Efficacy	95.95%	97.38%					98.61%		
A/Sw/Cotes d’Armor/0186/2010 (H1_av_N2)	Range of linearity^a^	<15.01 to 32.76	<15.01 to 32.76							<15.01 to 32.76
	Slope	−3.5616	−3.2659							−3.674
	R^2^	0.9992	0.9988							0.9962
	Efficacy	90.89%	102.39%							87.15%
A/Sw/Cotes d’Armor/0113/2006 (H1_hu_N2)	Range of linearity^a^	<14.52 to 28.49		<14.5 to 33.49						<14.52 to >33.49
	Slope	−3.637		−3.5244						−3.5025
	R^2^	0.9888		0.9938						0.9866
	Efficacy	88.34%		92.19%						92.98%
A/Sw/France/22–130212/2013 (H1_hu_N2_Δ146–147_)	Range of linearity^a^	<8.59 to >32.73		<8.59 to >25.44	<8.59 to >32.73					<8.59 to >32.73
	Slope	−3.4642		−3.5856	−3.6888					−3.6624
	R^2^	0.9989		0.9992	0.9979					0.9958
	Efficacy	94.39%		90.06%	86.68%					87.52%
A/Sw/Cotes d’Armor/0070/2010 (H1_hu_N1)	Range of linearity^a^	<14.85 to >29.68		<14.85 to 29.68				<14.85 to >29.68		
	Slope	−3.726		−3.307				−3.481		
	R^2^	0.9952		0.9903				0.995		
	Efficacy	85.52%		100.63%				93.76%		
A/Sw/Cotes d’Armor/0190/2006 (H1_hu_N1_Δ147_)	Range of linearity^a^	<13.49 to 31.31		<13.49 to 25.01				<13.49 to >31.31		
	Slope	−3.6041		−3.724				−3.6323		
	R^2^	0.9979		0.9958				0.9984		
	Efficacy	89.44%		85.58%				88.50%		
A/Sw/Sarthe/0255/2010 (H1N1pdm)	Range of linearity^a^	<13.31 to 33.50				<13.31 to 31.88		<13.31 to 31.88	<13.31 to 31.88	
	Slope	−3.5216				−3.4394		−3.4324	−3.589	
	R^2^	0.9927				0.9939		0.9887	0.9963	
	Efficacy	92.29%				95.32%		95.59%	89.95%	
A/Sw/Flandres/1/1998 (H3N2)	Range of linearity^a^	<12.96 to >34.01					<12.96 to >34.01			<12.96 to 30.48
	Slope	−3.485					−3.5005			−3.8987
	R^2^	0.9977					0.9931			0.9942
	Efficacy	93.62%					93.05%			80.51%

^a^The range of linearity is given as the interval of Cq-values obtained from M gene RT-qPCR on diluted samples. *R*^*2*^ Coefficient of linear regression

### Robustness of the RT-qPCR subtyping tool in low- and high-throughput analyses

The full RT-qPCR subtyping tool began to be used routinely by the French NRL for Swine Influenza from January 2014, as part of the analytical workflow for swIAV surveillance. Thus, nasal swab supernatants previously selected as containing M gene from a swIAV were subjected to H1_av_, H1_hu_ and H3 monoplex assays, to the N1/N2 duplex assay, as well as to commercial H1pdm and N1pdm RT-PCRs. Samples that were found to be positive for H1_hu_ were subjected to the H1_huΔ146–147_ RT-PCR in a second step. Considering the detection limits evaluated above, subtyping was undertaken on samples with M gene Cq-values <35 only. Samples exhibiting M gene Cq-values >35 were considered to be not typable. Looking at the proportions of HA and NA genes that were successfully subtyped in these samples from January 2014 to August 2016, it appeared that both genes were identified in almost all samples with M gene Cq-value <25 (Table 9). For 25 ≤ Cq-value <30, the proportion of unidentified HA and/or NA genes increased slightly. In these samples, the N1/N2 multiplex appeared less sensitive than HA simplex assays, as NA genes were less frequently amplified. In samples with Cq-value >30, the proportion of viruses fully or partially subtyped fell to about 50%.

**Table 9:** Percentages of HA and NA genes identified in nasal swab supernatants with M gene Cq-value <35, either in low throughput analyses (routine diagnosis performed by the French NRL, January 2014–August 2016) or in high-throughput analyses (longitudinal survey in three herds), according to the M gene Cq-values

Low-throughput analyses^a^				High-throughput analyses^b^			
Range of M gene Cq-values	Number of samples	Proportion of HA identified	Proportion of NA identified	Range of M gene Cq-values	Number of samples	Proportion of HA identified	Proportion of NA identified
30 ≤ Cq < 35	87	52.88%	20.56%	30 ≤ Cq < 33	189	12.17%	33.86%
25 ≤ Cq < 30	151	93.22%	79.19%	25 ≤ Cq < 30	273	89.38%	96.34%
20 ≤ Cq < 25	198	97.00%	99.14%	20 ≤ Cq < 25	350	98.86%	100%
< 20	106	100%	99.22%	< 20	106	100%	100%

^a^Samples tested for H1_av_, H1_hu_, H1pdm, H3, N1, N1pdm and N2. ^b^Samples tested for H1_av_, H1_hu_, N1 and N2 only as herds were previously known to be affected solely sby H1_av_N1 and H1_hu_N2 viruses

Finally, the diagnostic ability of the high-throughput subtyping RT-qPCR assay was tested on RNAs extracted from 919 nasal swabs collected during a longitudinal study conducted in three pig herds located in Brittany and previously selected as M gene-positive samples [28]. These herds were known to be affected by H1_av_N1 and H1_hu_N2 viruses only. Among them, HA and NA genes were both identified in 697 samples, whereas only the HA or NA gene was detected in 77 others, depending on the apparent amount of virus genome in the samples (Table 9). Almost all samples with an M gene Cq-value <30 were fully subtyped (Table 9). In samples with an M gene Cq-value >30, the NA gene was more frequently amplified than the HA gene (Table 9). Fourteen samples from one herd were shown to contain virus mixtures and/or reassortant viruses (data not shown).

## Discussion

Eight real-time RT-PCRs were evaluated for European swIAV molecular subtyping in clinical samples demonstrated to contain IAV genome. They aimed to discriminate HA genes for four H1 genetic lineages (H1_av_, H1_hu_, H1_huΔ146–147_, H1pdm) and one H3 lineage, as well as NA genes of two N1 lineages (N1, N1pdm) and one N2 lineage. Altogether, the RT-qPCRs enabled the identification of swIAVs from the four viral subtypes known to be enzootic in European pigs, i.e. H1_av_N1, H1_hu_N2, H3N2 and H1N1pdm. They also made it possible to quickly identify a new antigenic variant (H1_hu_N2_Δ146–147_) among H1_hu_N2 viruses, as well as reassortant viruses, i.e. H1_hu_N1 or H1_av_N2, and virus mixtures. All assays were optimized to take into account the genetic diversity encountered among swIAVs isolated in France in recent years, whereas keeping in mind usefulness on an European level. They exhibited a gain in sensitivity as compared to conventional RT-PCRs, allowing the characterization of biological samples with low genetic loads, with considerable time saving. Reagents and amplification procedures were harmonized to run all assays in parallel, using a unique amplification cycle profile irrespective of the equipment.

Evaluation in low-throughput analyses showed perfect specificities for H1_av_, H1_hu_ and H3 RT-qPCRs conducted in parallel as simplex assays. While multiplexing may facilitate the diagnostic procedure, preliminary studies showed that individual RT-qPCR sensitivities were somewhat affected when run in a triplex assay (data not shown). Based on M gene Cq-values, RT-qPCRs appeared slightly less sensitive than assays involving MGB-labelled probes, such as previously validated commercial kits or other in-house methods aimed at amplifying the M, H1pdm or N1pdm genes [21]. Nevertheless, the detection levels were satisfactory as HA subtyping was nearly 100% successful in clinical samples up to M gene Cq-value = 30. The H1_hu_ RT-qPCR was the least efficient, probably due to higher genetic variability among H1_hu_ genes, as compared to the H1_av_ and H3 genes [9]. The H1_huΔ146–147_ RT-qPCR, run in a second step on H1_hu_Ny-positive samples also demonstrated excellent specificity and sensitivity, leading to rapid discrimination of novel antigenic variants among H1_hu_N2 viruses. While H1_hu_N1 reassortants are rare events in most European countries, they are sporadically detected in France [8, 29] and some of them may also have an H1_huΔ146–147_ gene. In this study, two H1_hu_N1 reassortants exhibiting 2 amino acid deletions at positions 146–147 of the RBS, thus bearing H1 genes genetically and antigenically closer to the H1_huΔ146–147_ variant than to the parental H1_hu_, were not detected by the H1_huΔ146–147_ RT-PCR due to a mismatch within the “H1_hu__var” MGB probe. As a result, classification of the H1_hu_ gene from H1_hu_N1 reassortants as a “Δ146–147 variant” (or not) would be better confirmed by HA sequencing.

N1 and N2 RT-qPCRs, adapted from protocols previously evaluated at the European level, confirmed the specificities expected from in silico analyses [20]. While designed to amplify N2 genes from both European H1_hu_N2 and H3N2 lineages, the N2 RT-PCR proved to detect N2 genes from the novel H1_hu_N2_Δ146–147_ variants. By contrast to HA RT-qPCRs, they were run in a duplex assay because preliminary studies indicated no reduction in sensitivity as compared to corresponding simplex assays (data not shown). Nevertheless, it should be noted that in routine diagnosis, the NA gene was less frequently subtyped than the HA gene in samples with an M gene Cq-value >30, leading to the hypothesis that this difference could be related, at least partially, to multiplexing.

Scaling M and β-actin RT-qPCRs as well as HA (H1_av_, H1_hu_, H1_huΔ146–147_, H1pdm, H3) and NA (N1, N1pdm, N2) subtyping assays to higher throughput, with concurrent miniaturization of individual reactions, was successful. The LightCycler®1536 system designed for automated high-throughput laboratory workflows proved in this context to provide very high well-to-well homogeneity, good inter-assay reproducibility and low inter-plate variability. The 10 RT-qPCRs, run together onto one microplate using the same amplification procedure, showed very good efficiency. Each RT-qPCR retained its specificity. They all exhibited comparable and acceptable sensitivities. When run on clinical samples with an M gene Cq-value <30, the techniques were able to fully subtype more than 90% of detected swIAV genomes, showing an increased proportion of characterized NA genes in samples with Cq-values of 25–30 as compared to equivalent samples using classical thermocyclers. By contrast, when run on clinical samples with an M gene Cq-value >30, H1_av_, H1_hu_, N1 and N2 RT-PCRs appeared slightly less efficient than in low-throughput analyses. This could be related to the low volume of RNA extract included into the RT-qPCR mixture when run on LightCycler®1536 system as compared to other thermocyclers (1 μL instead of 5 μL), but this would need to be further investigated and confirmed for other subtyping RT-qPCRs.

## Conclusion

The emergence of the pandemic A/H1N1 virus of swine origin in 2009 highlighted the need for global surveillance of influenza A viruses in pigs. Its subsequent introduction into the pig population, its co-circulation with other enzootic swIAVs, and genomic reassortment events have led to an increase in the genetic diversity of swIAVs. Altogether, these RT-qPCR assays provide a rapid and simple genotyping method to identify viruses that infect the European pig population, including a novel H1_hu_N2 variant identified in France, sporadic HA/NA reassortants and virus mixtures, as a first characterization step before full genome sequencing and/or antigenic subtyping. Screening of individual samples against the 10 target genes in a high-throughput scenario opens novel perspectives in diagnostic abilities, which will be very useful for swIAV surveillance and large-scale epidemiological studies.

## Additional files

Additional file 1: Specificity of the real-time RT-PCR developed to identify H1_huΔ146–147_ antigenic variants among H1_hu_N_Y_ swIAVs (panel 2). (DOCX 18 kb)

Additional file 2: Identification of new antigenic variants among H1_hu_N_Y_ swIAVs by real-time RT-PCR targeting the H1_huΔ146–147_ gene. (DOCX 22 kb)

Additional file 3: Specificity of real-time RT-PCRs for detection and subtyping of swIAVs when run simultaneously as simplex assays on LightCycler^®^1536. (DOCX 61 kb)

## References

[1] Van Reeth K, Brown IH, Olsen CW. Influenza Virus. In: Zimmerman JJ, KLA, Ramirez A, Schwartz KJ, Stevenson GW, editors. Diseases of Swine. Tenth Edition. Wiley; 2012. p. 557-71.

[2] Dawood FS, Jain S, Finelli L, Shaw MW, Lindstrom S, Garten RJ, Gubareva LV, Xu X, Bridges CB, Uyeki TM (2009). Emergence of a novel swine-origin influenza a (H1N1) virus in humans. New Eng J Med.

[3] Myers KP, Olsen CW, Gray GC (2007). Cases of swine influenza in humans: a review of the literature. Clin Infect Dis.

[4] Cheung TK, Poon LL (2007). Biology of influenza a virus. Ann N Y Acad Sci.

[5] Nelson MI, Holmes EC (2007). The evolution of epidemic influenza. Nat Rev Genet.

[6] Webster RG, Bean WJ, Gorman OT, Chambers TM, Kawaoka Y (1992). Evolution and ecology of influenza a viruses. Microbiol Rev.

[7] Kuntz-Simon G, Madec F (2009). Genetic and antigenic evolution of swine influenza viruses in Europe and evaluation of their zoonotic potential. Zoonoses Public Health.

[8] Simon G, Larsen LE, Durrwald R, Foni E, Harder T, Van Reeth K, Markowska-Daniel I, Reid SM, Dan A, Maldonado J (2014). European surveillance network for influenza in pigs: surveillance programs, diagnostic tools and swine influenza virus subtypes identified in 14 European countries from 2010 to 2013. PLoS One.

[9] Watson SJ, Langat P, Reid SM, Lam TT, Cotten M, Kelly M, Van Reeth K, Qiu Y, Simon G, Bonin E (2015). Molecular epidemiology and evolution of influenza viruses circulating within European swine between 2009 and 2013. J Virol.

[10] Anderson TK, Macken CA, Lewis NS, Scheuermann RH, Van Reeth K, Brown IH, Swenson SL, Simon G, Saito T, Berhane Y, et al. A phylogeny-based global nomenclature system and automated annotation tool for H1 hemagglutinin genes from swine influenza a viruses. mSphere. 2016;1(6):e00275-16. 10.1128/mSphere.00275-16 PMC5156671 27981236

[11] Castrucci MR, Campitelli L, Ruggieri A, Barigazzi G, Sidoli L, Daniels R, Oxford JS, Donatelli I (1994). Antigenic and sequence analysis of H3 influenza virus haemagglutinins from pigs in Italy. J Gen Virol..

[12] Brown IH, Harris PA, McCauley JW, Alexander DJ (1998). Multiple genetic reassortment of avian and human influenza a viruses in European pigs, resulting in the emergence of an H1N2 virus of novel genotype. J Gen Virol..

[13] Smith GJ, Vijaykrishna D, Bahl J, Lycett SJ, Worobey M, Pybus OG, Ma SK, Cheung CL, Raghwani J, Bhatt S (2009). Origins and evolutionary genomics of the 2009 swine-origin H1N1 influenza a epidemic. Nature.

[14] Trebbien R, Bragstad K, Larsen LE, Nielsen J, Botner A, Heegaard PM, Fomsgaard A, Viuff B, Hjulsager CK (2013). Genetic and biological characterisation of an avian-like H1N2 swine influenza virus generated by reassortment of circulating avian-like H1N1 and H3N2 subtypes in Denmark. Virology J.

[15] Lange J, Groth M, Schlegel M, Krumbholz A, Wieczorek K, Ulrich R, Koppen S, Schulz K, Appl D, Selbitz H-J (2013). Reassortants of the pandemic (H1N1) 2009 virus and establishment of a novel porcine H1N2 influenza virus, lineage in Germany. Vet Microbiol.

[16] Moreno A, Chiapponi C, Boniotti MB, Sozzi E, Foni E, Barbieri I, Zanoni MG, Faccini S, Lelli D, Cordioli P (2012). Genomic characterization of H1N2 swine influenza viruses in Italy. Vet Microbiol.

[17] Moreno A, Gabanelli E, Sozzi E, Lelli D, Chiapponi C, Ciccozzi M, Zehender G, Cordioli P (2013). Different evolutionary trends of swine H1N2 influenza viruses in Italy compared to European viruses. Vet Res.

[18] Garin E, Hervé S, Rose N, Locatelli C, Ngwa-Mbot D, Wendling S, Bournez L, Calavas D, Simon G (2016). French network for the surveillance of influenza a viruses in pigs (Résavip) - results of the surveillance carried out in 2015. Bull Epid Santé Anim Alim.

[19] Chiapponi C, Moreno A, Barbieri I, Merenda M, Foni E (2012). Multiplex RT-PCR assay for differentiating European swine influenza virus subtypes H1N1, H1N2 and H3N2. J Virol Methods.

[20] Henritzi D, Zhao N, Starick E, Simon G, Krog JS, Larsen LE, Reid SM, Brown IH, Chiapponi C, Foni E (2016). Rapid detection and subtyping of European swine influenza viruses in porcine clinical samples by haemagglutinin- and neuraminidase-specific tetra- and triplex real-time RT-PCRs. Influenza Other Resp Viruses.

[21] Pol F, Quéguiner S, Gorin S, Deblanc C, Simon G (2011). Validation of commercial real-time RT-PCR kits for detection of influenza a viruses in porcine samples and differentiation of pandemic (H1N1) 2009 virus in pigs. J Virol Methods.

[22] OIE. Influenza A virus of swine. In: Manual of diagnostic tests and vaccines for terrestrial animals; 2015. Chapter 2.8.7. http://www.oie.int/fileadmin/Home/fr/Health_standards/tahm/2.08.07_INF_A_SWINE.pdf.

[23] Kyriakis CS, Brown IH, Foni E, Kuntz-Simon G, Maldonado J, Madec F, Essen SC, Chiapponi C, Van Reeth K (2011). Virological surveillance and preliminary antigenic characterization of influenza viruses in pigs in five European countries from 2006 to 2008. Zoonoses Public Health.

[24] Lewis NS, Russell CA, Langat P, Anderson TK, Berger K, Bielejec F, Burke DF, Dudas G, Fonville JM, Fouchier RA (2016). The global antigenic diversity of swine influenza a viruses. elife.

[25] Hoffmann E, Stech J, Guan Y, Webster RG, Perez DR (2001). Universal primer set for the full-length amplification of all influenza a viruses. Arch Virol.

[26] Kibbe WA (2007). OligoCalc: an online oligonucleotide properties calculator. Nucleic Acids Res.

[27] Deblanc C, Delgado-Ortega M, Gorin S, Berri M, Paboeuf F, Berthon P, Herrler G, Meurens F, Simon G (2016). Mycoplasma hyopneumoniae does not affect the interferon-related anti-viral response but predisposes the pig to a higher inflammation following swine influenza virus infection. J Gen Virol.

[28] Rose N, Hervé S, Eveno E, Barbier N, Eono F, Dorenlor V, Andraud M, Camsusou C, Madec F, Simon G (2013). Dynamics of influenza a virus infections in permanently infected pig farms: evidence of recurrent infections, circulation of several swine influenza viruses and reassortment events. Vet Res.

[29] Simon G, Hervé S, Rose N (2013). Epidemiosurveillance of swine influenza in France from 2005 to 2012: programs, viruses and associated epidemiological data. Bull Epid Santé Anim Alim.

[30] Weingartl HM, Berhane Y, Hisanaga T, Neufeld J, Kehler H, Emburry-Hyatt C, Hooper-McGreevy K, Kasloff S, Dalman B, Bystrom J (2010). Genetic and pathobiologic characterization of pandemic H1N1 2009 influenza viruses from a naturally infected swine herd. J Virol.

[31] Duvigneau JC, Hartl RT, Groiss S, Gemeiner M (2005). Quantitative simultaneous multiplex real-time PCR for the detection of porcine cytokines. J Immunol Methods.

