# Supplementary material for: Molecular subtyping of European swine influenza viruses and scaling to high-throughput analysis
Source: Virol J. 2018 Jan 10;15:7. doi: 10.1186/s12985-018-0920-z (PMC5761149; doi:10.1186/s12985-018-0920-z)
Supplement: Supplementary file 1 — Specificity of the real-time RT-PCR developed to identify H1huΔ146–147 antigenic variants among H1huNY swIAVs (panel 2). (DOCX 18 kb) [file 12985_2018_920_MOESM1_ESM.docx]

**Additional file 1.** Specificity of the real-time RT-PCR developed to identify H1_huΔ146-147_ antigenic variants among H1_hu_N_Y_ swIAVs.

| **Virus strains** (panel 2) | | | **H1_huΔ146-147_ real-time RT-PCR** (Cq-value) |
| --- | --- | --- | --- |
| **Subtype and lineage** (known from molecular subtyping) | **HA identity** (known from sequencing) | **Name** |  |
| H1_hu_N2 | H1_hu_ | A/Sw/France/29-110415/11 | no Cq |
|  |  | A/Sw/France/22-120049/12 | no Cq |
|  |  | A/Sw/France/22-120051/12 | no Cq |
|  |  | A/Sw/France/22-120228/12 | no Cq |
|  |  | A/Sw/France/22-120334/12 | no Cq |
|  |  | A/Sw/France/22-120424/12 | no Cq |
|  |  | A/Sw/France/29-120364/12 | no Cq |
|  |  | A/Sw/France/22-120390/12 | no Cq |
|  |  | A/Sw/France/22-120391/12 | no Cq |
|  |  | A/Sw/France/56-120456/12 | no Cq |
|  |  | A/Sw/France/35-130024/13 | no Cq |
|  |  | A/Sw/France/22-130078/13 | no Cq |
|  |  | A/Sw/France/22-130277/13 | no Cq |
|  |  | A/Sw/France/29-130429/13 | no Cq |
|  |  | A/Sw/France/29-140033/14 | no Cq |
|  |  | A/Sw/France/29-140137/14 | no Cq |
|  |  | A/Sw/France/29-140286/14 | no Cq |
|  |  | A/Sw/France/29-140342/14 | no Cq |
|  |  | A/Sw/France/56-140511/14 | no Cq |
|  | H1_huΔ146-147_ | A/Sw/France/35-120245/12 | 14.80 |
|  |  | A/Sw/France/56-120285/12 | 15.01 |
|  |  | A/Sw/France/35-120354/12 | 14.21 |
|  |  | A/Sw/France/22-120355/12 | 15.59 |
|  |  | A/Sw/France/56-120356/12 | 15.65 |
|  |  | A/Sw/France/22-120417/12 | 15.41 |
|  |  | A/Sw/France/35-130023/13 | 15.94 |
|  |  | A/Sw/France/22-130058/13 | 14.23 |
|  |  | A/Sw/France/22-130083/13 | 13.54 |
|  |  | A/Sw/France/22-130103/13 | 14.67 |
|  |  | A/Sw/France/22-130140/13 | 16.77 |
|  |  | A/Sw/France/22-130176/13 | 15.33 |
|  |  | A/Sw/France/22-130212/13 | 19.58 |
|  |  | A/Sw/France/35-130431/13 | 16.39 |
|  |  | A/Sw/France/22-140027/14 | 20.47 |
|  |  | A/Sw/France/50-140056/14 | 15.32 |
|  |  | A/Sw/France/50-140058/14 | 13.22 |
|  |  | A/Sw/France/22-140062/14 | 16.09 |
|  |  | A/Sw/France/22-140075/14 | 13.92 |
|  |  | A/Sw/France/22-140102/14 | 18.43 |
|  |  | A/Sw/France/29-140221/14 | 14.41 |
|  |  | A/Sw/France/22-140256/14 | 23.35 |
|  |  | A/Sw/France/22-140494/14 | 15.46 |
|  |  | A/Sw/France/22-140496/14 | 18.44 |
|  |  | A/Sw/France/22-140510/14 | 20.45 |
| H1_hu_N1 | H1_huΔ146-147_ | A/Sw/Cotes d'Armor/0619/11 | no Cq |
|  |  | A/Sw/France/22-120067/12 | no Cq |
