# Supplementary material for: Molecular subtyping of European swine influenza viruses and scaling to high-throughput analysis
Source: Virol J. 2018 Jan 10;15:7. doi: 10.1186/s12985-018-0920-z (PMC5761149; doi:10.1186/s12985-018-0920-z)
Supplement: Supplementary file 2 — Identification of new antigenic variants among H1huNY swIAVs by real-time RT-PCR targeting the H1huΔ146–147 gene. (DOCX 22 kb) [file 12985_2018_920_MOESM2_ESM.docx]

**Additional file 2.** Identification of new antigenic variants among H1_hu_N_Y_ swIAVs by real-time RT-PCR targeting the H1_huΔ146-147_ gene.

| **Samples** (panel 4) | | | | **H1_huΔ146-147_**  **real-time RT-PCR**  (Cq-value) |
| --- | --- | --- | --- | --- |
| **Type** | **swIAV lineage known from conventional RT-PCRs and commercial H1pdm and N1pdm RT-qPCRs** | | **Strain name or sample identification** |  |
|  | **HA** | **NA** |  |  |
| Viral isolate | H1_hu_ | N2 | A/Sw/Cotes d'Armor/0028/11 | no Cq |
|  |  |  | A/Sw/France/29-110226/11 | no Cq |
|  |  |  | A/Sw/Ille et Vilaine/0399/11 | no Cq |
|  |  |  | A/Sw/Cotes d'Armor/0544/11 | no Cq |
|  |  |  | A/Sw/Cotes d'Armor/0569/11 | no Cq |
|  |  |  | A/Sw/Maine et Loire/0589/11 | no Cq |
|  |  |  | A/Sw/Finistere/0608/11 | no Cq |
|  |  |  | A/Sw/Cotes d'Armor/0636/11 | no Cq |
|  |  |  | A/Sw/France/22-110620/11 | no Cq |
|  |  |  | A/Sw/France/56-130420/13 | no Cq |
|  |  |  | A/Sw/France/53-140045/13 | 12.37 |
|  |  |  | A/Sw/France/29-150034/15 | no Cq |
|  |  |  | A/Sw/France/56-150097/15 | 19.81 |
|  |  |  | A/Sw/France/29-150218/15 | no Cq |
|  |  |  | A/Sw/France/22-150235/15 | 13.86 |
|  | H1_hu_/H1_av_ | N2 | 120258-6/P1/MDCK | no Cq |
|  |  |  | 130012-1/P1/MDCK | no Cq |
|  |  |  | 130133-2/P1/MDCK | no Cq |
|  |  | N1/N2 | 140468-1/P1/MDCK | no Cq |
|  |  | N1 | 140374-3/P1/MDCK | no Cq |
|  |  |  | 140399-3/P1/MDCK | no Cq |
|  |  |  | 140468-2/P1/MDCK | no Cq |
| Nasal swab supernatants | H1_hu_ | N2 | 110486-3 | no Cq |
|  |  |  | 110491-2 | no Cq |
|  |  |  | 110493-3 | no Cq |
|  |  |  | 110530-3 | no Cq |
|  |  |  | 110503-1 | no Cq |
|  |  |  | 110504-1 | no Cq |
|  |  |  | 110416 | no Cq |
|  |  |  | 120042 | no Cq |
|  |  |  | 120046 | no Cq |
|  |  |  | 120004 | no Cq |
|  |  |  | 120037-1 | no Cq |
|  |  |  | 120039 | no Cq |
|  |  |  | 120040 | no Cq |
|  |  |  | 120041 | no Cq |
|  |  |  | 120129 | no Cq |
|  |  |  | 130026 | 21.55 |
|  |  |  | 130039-1 | no Cq |
|  |  |  | 130059-1 | no Cq |
|  |  |  | 130162-3 | 24.47 |
|  |  |  | 130193-1 | no Cq |
|  |  |  | 130232-1 | no Cq |
|  |  |  | 130246-2 | no Cq |
|  |  |  | 130280-2 | 17.83 |
|  |  |  | 130348-2 | 23.2 |
|  |  |  | 130368-3 | 21.07 |
|  |  |  | 130437-1 | 21.62 |
|  |  |  | 130438-2 | 22.01 |
|  |  |  | 140403-4 | 36.67 |
|  |  |  | 140436-4 | no Cq |
|  |  |  | 150024-4 | no Cq |
|  |  |  | 150031-4 | 30.46 |
|  |  |  | 150038-4 | 31.47 |
|  |  |  | 150071-4 | 31.05 |
|  |  |  | 150077-4 | no Cq |
|  |  |  | 150081-4 | no Cq |
|  |  |  | 150082-4 | 35.96 |
|  |  |  | 150140-4 | 36.4 |
|  |  |  | 150176-5 | 23.64 |
|  |  |  | 150192-3 | 24.16 |
|  |  |  | 150231-4 | no Cq |
|  |  |  | 150237-4 | no Cq |
|  |  |  | 150238-6 | no Cq |
|  |  |  | 150250-6 | no Cq |
|  |  |  | 150254-6 | 27.46 |
|  |  |  | 150258-4 | no Cq |
|  |  |  | 150314-2 | no Cq |
|  | H1_hu_ | N? | 130234-1 | no Cq |
|  |  |  | 140223-1 | no Cq |
|  |  |  | 140287-2 | no Cq |
|  |  |  | 140344-5 | 33.99 |
|  |  |  | 150053-2 | no Cq |
|  |  |  | 150201-4 | no Cq |
|  | H1_hu_/H1_av_ | N1 | 130044 | no Cq |
|  | H? | N2 | 120071 | no Cq |
|  | H? | N? | 110527-3 | no Cq |
|  | H? | N? | 110506-3 | no Cq |
