# Supplementary material for: Molecular subtyping of European swine influenza viruses and scaling to high-throughput analysis
Source: Virol J. 2018 Jan 10;15:7. doi: 10.1186/s12985-018-0920-z (PMC5761149; doi:10.1186/s12985-018-0920-z)
Supplement: Supplementary file 3 — Specificity of real-time RT-PCRs for detection and subtyping of swIAVs when run simultaneously as simplex assays on LightCycler®1536. (DOCX 61 kb) [file 12985_2018_920_MOESM3_ESM.docx]

**Additional file 3.** Specificity of real-time RT-PCRs for detection and subtyping of swIAVs when run simultaneously as simplex assays on LightCycler^®^1536.

| **Samples** (panel 5) | | | | **Real-time RT-PCR** (Cq-value) | | | | | | | | | |
| --- | --- | --- | --- | --- | --- | --- | --- | --- | --- | --- | --- | --- | --- |
| **Sample type** | **Subtype and lineage** | | **Strain name or sample identification** |  |  |  |  |  |  |  |  |  |  |
|  |  |  |  | **M** | **β-actin** | **H1_av_** | **H1_hu_** | **H1_huΔ146-147_** | **H1_pdm_** | **H3** | **N1** | **N1_pdm_** | **N2** |
| Virus isolate | H1_av_N1 | | A/Sw/Côtes d'Armor/0388/2009 | 7.69 | 17.57 | 8.85 | no Cq | no Cq | no Cq | no Cq | 8.68 | no Cq | no Cq |
|  |  |  | A/Sw/France/29-130398/2013 | 6.63 | 22.26 | 8.53 | no Cq | no Cq | no Cq | no Cq | 10.10 | no Cq | no Cq |
|  |  |  | A/Sw/France/56-130007/2013 | 11.96 | 24.81 | 14.12 | no Cq | no Cq | no Cq | no Cq | 14.82 | no Cq | no Cq |
|  |  |  | A/Sw/France/50-120350/2012 | 5.23 | 20.94 | 8.40 | no Cq | no Cq | no Cq | no Cq | 8.96 | no Cq | no Cq |
|  |  |  | A/Sw/France/79-140150/2014 | 12.05 | **no Cq** | 13.47 | no Cq | no Cq | no Cq | no Cq | 14.57 | no Cq | no Cq |
|  |  |  | A/Sw/France/89-150069/2015 | 9.55 | 21.70 | 9.27 | no Cq | no Cq | no Cq | no Cq | 11.28 | no Cq | no Cq |
|  |  |  | A/Sw/France/72-140149/2014 | 9.55 | 21.66 | 13.49 | no Cq | no Cq | no Cq | no Cq | 10.13 | no Cq | no Cq |
|  |  |  | A/Sw/France/79-140150/2014 | 7.00 | 21.76 | 8.94 | no Cq | no Cq | no Cq | no Cq | 10.33 | no Cq | no Cq |
|  |  |  | A/Sw/France/56-130134/2013 | 9.33 | 20.64 | 10.07 | no Cq | no Cq | no Cq | no Cq | 13.18 | no Cq | no Cq |
|  |  |  | A/Sw/France/59-130139/2013 | 7.28 | 19.90 | 11.79 | no Cq | no Cq | no Cq | no Cq | 3.24 | no Cq | no Cq |
|  |  |  | A/Sw/France/01-130145/2013 | 16.69 | 22.67 | 16.59 | no Cq | no Cq | no Cq | no Cq | 21.79 | no Cq | no Cq |
|  |  |  | A/Sw/France/22-130153/2013 | 10.32 | 17.45 | 19.25 | no Cq | no Cq | no Cq | no Cq | 15.40 | no Cq | no Cq |
|  |  |  | A/Sw/France/56-130163/2013 | 7.44 | 19.57 | 9.37 | no Cq | no Cq | no Cq | no Cq | 9.47 | no Cq | no Cq |
|  |  |  | A/Sw/France/22-130166/2013 | < 5 | 22.81 | 7.10 | no Cq | no Cq | no Cq | no Cq | 2.92 | no Cq | no Cq |
|  |  |  | A/Sw/France/59-130174/2013 | 13.51 | 26.61 | 15.24 | no Cq | no Cq | no Cq | no Cq | 14.05 | no Cq | no Cq |
|  | H1_av_N2 | | A/Sw/Côtes d'Armor/0186/2010 | 11.75 | 30.59 | 14.51 | no Cq | no Cq | no Cq | no Cq | no Cq | no Cq | 11.54 |
|  |  |  | A/Sw/Côtes d'Armor/0102/2008-E | 13.49 | 30.60 | 13.43 | no Cq | no Cq | no Cq | no Cq | no Cq | no Cq | 12.98 |
|  |  |  | A/Sw/Finistere/0181/2010 | 7.24 | 21.54 | 10.20 | no Cq | no Cq | no Cq | no Cq | no Cq | no Cq | 7.17 |
|  |  |  | A/Sw/France/22-110529/2011 | 11.90 | 31.40 | 13.50 | no Cq | no Cq | no Cq | no Cq | no Cq | no Cq | 13.58 |
|  |  |  | A/Sw/Morbihan/0213/2011 | 7.69 | 22.42 | 11.14 | no Cq | no Cq | no Cq | no Cq | no Cq | no Cq | < 5 |
|  |  |  | A/Sw/Morbihan/0599/2011 | 10.56 | 15.72 | 13.63 | no Cq | no Cq | no Cq | no Cq | no Cq | no Cq | 11.41 |
|  |  |  | A/Sw/France/22-120057/2012 | 11.58 | 27.88 | 13.33 | no Cq | no Cq | no Cq | no Cq | no Cq | no Cq | 12.13 |
|  |  |  | A/Sw/France/37-120345/2012 | 8.64 | 21.47 | 10.58 | no Cq | no Cq | no Cq | no Cq | no Cq | no Cq | < 5 |
|  |  |  | A/Sw/France/22-130133_3/2013 | 12.06 | 31.40 | 13.32 | no Cq | no Cq | no Cq | no Cq | no Cq | no Cq | 12.00 |
|  |  |  | A/Sw/France/61-140350/2014 | 6.28 | 20.45 | < 5 | no Cq | no Cq | no Cq | no Cq | no Cq | no Cq | 6.99 |
|  |  |  | A/Sw/France/64-150152/2015 | 10.44 | 21.12 | 11.25 | no Cq | no Cq | no Cq | no Cq | no Cq | no Cq | 10.22 |
|  | H1_hu_N2 | | A/Sw/Côtes d'Armor/0113/2006 | 10.39 | 30.02 | no Ct | 12.02 | no Ct | no Ct | no Ct | no Ct | no Ct | 11.25 |
|  | H1_hu_N2_Δ146-147_ | | A/Sw/France/22-130212/2013 | 6.18 | 20.63 | no Cq | 7.75 | < 5 | no Cq | no Cq | no Cq | no Cq | < 5 |
|  |  |  | A/Sw/France/35-120245/2012 | 7.78 | 23.09 | no Cq | 11.04 | 8.96 | no Cq | no Cq | no Cq | no Cq | 5.68 |
|  |  |  | A/Sw/France/22-120255/2012 | 6.66 | 21.50 | no Cq | 9.46 | 7.10 | no Cq | no Cq | no Cq | no Cq | < 5 |
|  |  |  | A/Sw/France/56-120285/2012 | 11.21 | **no Cq** | no Cq | 13.97 | 11.96 | no Cq | no Cq | no Cq | no Cq | 12.74 |
|  |  |  | A/Sw/France/35-120354/2012 | 6.55 | 21.55 | no Cq | < 5 | 6.34 | no Cq | no Cq | no Cq | no Cq | 8.62 |
|  |  |  | A/Sw/France/22-120355/2012 | 8.53 | 22.12 | no Cq | 11.37 | 10.49 | no Cq | no Cq | no Cq | no Cq | 7.37 |
|  |  |  | A/Sw/France/56-120356/2012 | 8.51 | 21.38 | no Cq | 11.44 | 10.46 | no Cq | no Cq | no Cq | no Cq | 10.17 |
|  |  |  | A/Sw/France/22-120417/2012 | 7.95 | 19.93 | no Cq | 11.83 | 9.58 | no Cq | no Cq | no Cq | no Cq | 9.42 |
|  |  |  | A/Sw/France/35-130023/2013 | 6.61 | 20.51 | no Cq | 10.54 | 7.50 | no Cq | no Cq | no Cq | no Cq | 8.18 |
|  |  |  | A/Sw/France/22-130032/2013 | 6.27 | 20.34 | no Cq | 10.62 | 8.13 | no Cq | no Cq | no Cq | no Cq | 5.09 |
|  |  |  | A/Sw/France/22-130034/2013 | 6.47 | 18.89 | no Cq | 10.62 | 7.28 | no Cq | no Cq | no Cq | no Cq | 9.24 |
|  |  |  | A/Sw/France/22-130058/2013 | < 5 | 19.99 | no Cq | 7.45 | 10.31 | no Cq | no Cq | no Cq | no Cq | 7.14 |
|  |  |  | A/Sw/France/22-130083/2013 | 6.26 | 21.88 | no Cq | 10.82 | 6.37 | no Cq | no Cq | no Cq | no Cq | < 5 |
|  |  |  | A/Sw/France/53-130111/2013 | 6.95 | 18.90 | no Cq | 9.43 | 8.09 | no Cq | no Cq | no Cq | no Cq | < 5 |
|  |  |  | A/Sw/France/22-130140/2013 | 9.07 | 22.98 | no Cq | 14.48 | 9.18 | no Cq | no Cq | no Cq | no Cq | 11.10 |
|  |  |  | A/Sw/France/22-130176/2013 | 7.93 | 19.40 | no Cq | 11.24 | 10.38 | no Cq | no Cq | no Cq | no Cq | 9.23 |
|  |  |  | A/Sw/France/35-130431/2013 | 8.61 | 23.11 | no Cq | 13.46 | 10.56 | no Cq | no Cq | no Cq | no Cq | 9.27 |
|  |  |  | A/Sw/France/53-140045/2013 | 10.60 | 21.51 | no Cq | 13.92 | 12.14 | no Cq | no Cq | no Cq | no Cq | 11.70 |
|  |  |  | A/Sw/France/22-140027/2014 | 10.08 | 21.05 | no Cq | 13.16 | 12.13 | no Cq | no Cq | no Cq | no Cq | 10.59 |
|  |  |  | A/Sw/France/35-140041/2014 | 8.04 | 19.23 | no Cq | 7.61 | 9.71 | no Cq | no Cq | no Cq | no Cq | < 5 |
|  |  |  | A/Sw/France/50-140056/2014 | 7.41 | 19.99 | no Cq | 9.66 | < 5 | no Cq | no Cq | no Cq | no Cq | < 5 |
|  |  |  | A/Sw/France/50-140058/2014 | 6.67 | 18.83 | no Cq | 6.66 | 6.77 | no Cq | no Cq | no Cq | no Cq | < 5 |
|  |  |  | A/Sw/France/22-140062/2014 | 7.96 | 22.42 | no Cq | 12.64 | 11.20 | no Cq | no Cq | no Cq | no Cq | 10.31 |
|  |  |  | A/Sw/France/22-140075/2014 | 7.54 | 19.12 | no Cq | 9.96 | 8.76 | no Cq | no Cq | no Cq | no Cq | < 5 |
|  |  |  | A/Sw/France/22-140102/2014 | 9.97 | 23.92 | no Cq | 12.80 | 11.32 | no Cq | no Cq | no Cq | no Cq | 11.53 |
|  |  |  | A/Sw/France/29-140221/2014 | 8.26 | 20.16 | no Cq | 11.51 | 10.01 | no Cq | no Cq | no Cq | no Cq | 6.14 |
|  |  |  | A/Sw/France/22-140256/2014 | 8.24 | 28.71 | no Cq | 11.16 | 8.77 | no Cq | no Cq | no Cq | no Cq | 9.41 |
|  |  |  | A/Sw/France/56-140486/2014 | 12.44 | 31.04 | no Cq | 15.29 | 13.30 | no Cq | no Cq | no Cq | no Cq | 13.52 |
|  |  |  | A/Sw/France/22-140494/2014 | 16.71 | 33.55 | no Cq | 17.61 | 17.76 | no Cq | no Cq | no Cq | no Cq | 15.69 |
|  |  |  | A/Sw/France/22-140496/2014 | 8.90 | 23.47 | no Cq | 11.89 | 13.21 | no Cq | no Cq | no Cq | no Cq | 8.79 |
|  |  |  | A/Sw/France/22-140510/2014 | 9.43 | 24.18 | no Cq | 12.78 | 10.79 | no Cq | no Cq | no Cq | no Cq | 8.82 |
|  |  |  | A/Sw/France/56-150097/2015 | 12.61 | 28.72 | no Cq | < 5 | 12.50 | no Cq | no Cq | no Cq | no Cq | 11.49 |
|  | H1_hu_N1 | | A/Sw/Côtes d'Armor/0070/2010 | 11.40 | 29.64 | no Cq | 14.96 | no Cq | no Cq | no Cq | 13.88 | no Cq | no Cq |
|  |  |  | A/Sw/Cotes d'Armor/060293/2001 | 11.76 | 25.50 | no Cq | 17.57 | no Cq | no Cq | no Cq | 15.17 | no Cq | no Cq |
|  |  |  | A/Sw/Cotes d'Armor/098574/2001 | 11.42 | 26.20 | no Cq | 16.94 | no Cq | no Cq | no Cq | 15.54 | no Cq | no Cq |
|  |  |  | A/Sw/Côtes d'Armor/0046/2008 | 9.53 | 22.63 | no Cq | 12.88 | no Cq | no Cq | no Cq | 12.14 | no Cq | no Cq |
|  | H1_hu_N1_Δ147_ | | A/Sw/Côtes d'Armor/0190/2006 | 10.21 | 21.23 | no Cq | 13.86 | no Cq | no Cq | no Cq | 12.94 | no Cq | no Cq |
|  | H1_hu_N1_Δ146-147_ | | A/Sw/Cotes d'Armor/0619/2011 | 13.73 | 29.86 | no Cq | 16.21 | no Cq | no Cq | no Cq | 15.20 | no Cq | no Cq |
|  |  |  | A/Sw/France/22-120067/2012 | 9.94 | 22.52 | no Cq | 12.78 | no Cq | no Cq | no Cq | 11.78 | no Cq | no Cq |
|  | H1N1_pdm_ | | A/Sw/Sarthe/0255/2010 | 10.37 | 27.94 | no Cq | no Cq | no Cq | 14.51 | no Cq | 14.80 | 12.96 | no Cq |
|  |  |  | A/California/04/2009 | 8.96 | 27.84 | no Cq | no Cq | no Cq | 11.13 | no Cq | 11.86 | 11.72 | no Cq |
|  |  |  | A/Cat/France/0514/2009 | 11.25 | 29.94 | no Cq | no Cq | no Cq | 14.56 | no Cq | 14.45 | 13.92 | no Cq |
|  |  |  | A/Sw/Cotes d'Armor/110466/2010 | 8.03 | 19.86 | no Cq | no Cq | no Cq | 11.85 | no Cq | 12.04 | 11.71 | no Cq |
|  |  |  | A/Sw/Haute-Loire/0578/2011 | 7.19 | 19.32 | no Cq | no Cq | no Cq | 9.96 | no Cq | 10.63 | 9.14 | no Cq |
|  |  |  | A/Sw/France/18-120333/2012 | 9.82 | 20.80 | no Cq | no Cq | no Cq | 12.74 | no Cq | 12.99 | 12.77 | no Cq |
|  |  |  | A/Sw/France/71-130116/2013 | 13.12 | 29.93 | no Cq | no Cq | no Cq | 17.36 | no Cq | 14.71 | 16.42 | no Cq |
|  |  |  | A/Sw/France/57-140136/2014 | 8.02 | 21.92 | no Cq | no Cq | no Cq | 11.61 | no Cq | 10.88 | 11.49 | no Cq |
|  |  |  | A/Sw/France/35-140382/2014 | 7.52 | 21.21 | no Cq | no Cq | no Cq | 12.02 | no Cq | 11.66 | 11.78 | no Cq |
|  |  |  | A/Sw/France/35-140384/2014 | 7.55 | 19.87 | no Cq | no Cq | no Cq | 11.11 | no Cq | 10.93 | 11.21 | no Cq |
|  |  |  | A/Sw/France/64-150052/2015 | 10.71 | 22.56 | no Cq | no Cq | no Cq | 14.31 | no Cq | 15.04 | 15.64 | no Cq |
|  |  |  | A/Sw/France/12-150058/2015 | 9.89 | 21.05 | no Cq | no Cq | no Cq | 12.76 | no Cq | 14.18 | 14.26 | no Cq |
|  |  |  | A/Sw/France/64-150091/2015 | 8.54 | 19.80 | no Cq | no Cq | no Cq | 13.13 | no Cq | 11.96 | 12.93 | no Cq |
|  | H3N2 | | A/Sw/Flandres/1/1998 | 9.86 | 28.61 | no Cq | no Cq | no Cq | no Cq | 10.08 | no Cq | no Cq | 11.89 |
|  |  |  | A/Sw/France/59-120031/2012 | 10.67 | 29.27 | no Cq | no Cq | no Cq | no Cq | 12.00 | no Cq | no Cq | 10.20 |
|  |  |  | A/Sw/France/59-140441/2014 | 8.77 | 24.02 | no Cq | no Cq | no Cq | no Cq | 11.72 | no Cq | no Cq | 10.61 |
|  |  |  | A/Sw/France/62-140458/2014 | 10.30 | 23.76 | no Cq | no Cq | no Cq | no Cq | 12.87 | no Cq | no Cq | 12.28 |
| Virus mixtures in MDCK supernatants | | H1_av_/H1_hu_/N1_av_/N2 | 140374-3/P1/MDCK | 9.06 | 22.14 | 11.31 | 23.93 | no Cq | no Cq | no Cq | 11.97 | no Cq | no Cq |
|  |  |  | 140399-1/P1/MDCK | 13.34 | 29.50 | 14.28 | 22.02 | no Cq | no Cq | no Cq | 14.65 | no Cq | no Cq |
|  |  |  | 140468_2/P1/MDCK | 9.75 | 17.90 | 16.15 | 27.62 | no Cq | no Cq | no Cq | 14.03 | no Cq | no Cq |
|  |  | H1_av_/H1_hu_/ N1_av_ | 130133-2/P1/MDCK | 11.21 | 30.71 | 13.10 | 12.72 | no Cq | no Cq | no Cq | no Cq | no Cq | 11.23 |
|  |  | H1_av_/H1_hu_/N2 | 140468-1/P1/MDCK | 7.78 | 21.81 | 16.33 | 17.31 | no Cq | no Cq | no Cq | 11.75 | no Cq | **no Cq** |
| Nasal swab supernatants | | H1_av_N1 | 150070 | 21.62 | 21.83 | 24.16 | no Cq | no Cq | no Cq | no Cq | 23.39 | no Cq | no Cq |
|  |  |  | 150080 | 24.23 | 23.96 | 26.03 | no Cq | no Cq | no Cq | no Cq | 25.76 | no Cq | no Cq |
|  | |  | 150127 | 16.40 | 20.16 | 18.31 | no Cq | no Cq | no Cq | no Cq | 19.45 | no Cq | no Cq |
|  | |  | 150129 | 15.01 | 20.41 | 17.04 | no Cq | no Cq | no Cq | no Cq | 17.35 | no Cq | no Cq |
|  | |  | 150139 | 20.11 | 17.83 | 25.15 | no Cq | no Cq | no Cq | no Cq | 21.91 | no Cq | no Cq |
|  | |  | 150144 | 20.29 | 20.45 | 23.44 | no Cq | no Cq | no Cq | no Cq | 22.96 | no Cq | no Cq |
|  | |  | 140145 | 21.34 | 19.46 | 24.61 | no Cq | no Cq | no Cq | no Cq | 23.42 | no Cq | no Cq |
|  | |  | 140146 | 21.39 | 20.59 | 29.05 | no Cq | no Cq | no Cq | no Cq | 23.87 | no Cq | no Cq |
|  | |  | 140172 | 21.11 | 20.52 | 26.90 | no Cq | no Cq | no Cq | no Cq | 24.58 | no Cq | no Cq |
|  | |  | 130135 | 27.85 | 19.68 | 28.83 | no Cq | no Cq | no Cq | no Cq | 28.75 | no Cq | no Cq |
|  | |  | 130152 | 14.21 | 18.08 | 23.57 | no Cq | no Cq | no Cq | no Cq | 29.05 | no Cq | no Cq |
|  | |  | 130157 | 19.52 | 19.23 | 20.02 | no Cq | no Cq | no Cq | no Cq | 19.94 | no Cq | no Cq |
|  | |  | 130161 | 18.09 | 16.90 | 20.09 | no Cq | no Cq | no Cq | no Cq | 18.94 | no Cq | no Cq |
|  | |  | 130164 | 16.76 | 21.04 | 19.12 | no Cq | no Cq | no Cq | no Cq | 18.23 | no Cq | no Cq |
|  | | H1_av_N2 | 110214 | 29.88 | 22.55 | 31.13 | no Cq | no Cq | no Cq | no Cq | no Cq | no Cq | 30.53 |
|  | | H1_hu_N2_Δ146-147_ | 130162 | 24.25 | 25.07 | no Cq | 27.71 | 27.40 | no Cq | no Cq | no Cq | no Cq | 25.20 |
|  | |  | 130280 | 18.23 | 19.92 | no Cq | 21.46 | 20.67 | no Cq | no Cq | no Cq | no Cq | 17.82 |
|  | |  | 130348 | 21.07 | 21.77 | no Cq | 23.98 | 23.51 | no Cq | no Cq | no Cq | no Cq | 20.87 |
|  | |  | 130368 | 17.83 | 15.25 | no Cq | 21.66 | 20.22 | no Cq | no Cq | no Cq | no Cq | 19.25 |
|  | |  | 130437 | 19.52 | 22.27 | no Cq | 22.68 | 22.28 | no Cq | no Cq | no Cq | no Cq | 19.46 |
|  | |  | 130438 | 19.27 | 20.98 | no Cq | 22.29 | 21.27 | no Cq | no Cq | no Cq | no Cq | 18.48 |
|  | |  | 140403 | 20.95 | 17.87 | no Cq | 25.38 | 23.49 | no Cq | no Cq | no Cq | no Cq | 22.15 |
|  | |  | 150031 | 22.89 | 22.47 | no Cq | 26.17 | 25.29 | no Cq | no Cq | no Cq | no Cq | 23.98 |
|  | |  | 150038 | 21.20 | 21.17 | no Cq | 22.30 | 22.21 | no Cq | no Cq | no Cq | no Cq | 19.76 |
|  | |  | 150071 | 19.86 | 20.22 | no Cq | 21.93 | 18.78 | no Cq | no Cq | no Cq | no Cq | 16.67 |
|  | |  | 150082 | 19.63 | 19.07 | no Cq | 20.49 | 18.24 | no Cq | no Cq | no Cq | no Cq | 14.58 |
|  | |  | 150140 | 18.17 | 20.81 | no Cq | 22.65 | 20.57 | no Cq | no Cq | no Cq | no Cq | 18.30 |
|  | | H1N1pdm | 130233 | 20.54 | 21.02 | no Cq | no Cq | no Cq | 24.03 | no Cq | 23.24 | 23.40 | no Cq |
|  | |  | 150169 | 26.26 | 18.68 | no Cq | no Cq | no Cq | 29.41 | no Cq | 27.79 | 29.34 | no Cq |
|  | | H1_av_/H1_pdm/_N1_av_/N1_pdm_ | 150203 | 8.62 | 22.76 | 10.87 | no Cq | no Cq | 16.65 | no Cq | 10.87 | 14.18 | no Cq |

Data that do not correspond to expected results are highlighted in bold.
